# Genome Analysis of *Streptomyces nojiriensis* JCM 3382 and Distribution of Gene Clusters for Three Antibiotics and an Azasugar across the Genus *Streptomyces*

**DOI:** 10.3390/microorganisms9091802

**Published:** 2021-08-25

**Authors:** Jin-Soo Park, Da-Eun Kim, Sung-Chul Hong, Seung-Young Kim, Hak Cheol Kwon, Chang-Gu Hyun, Jaeyoung Choi

**Affiliations:** 1 Natural Product Informatics Research Center, Korea Institute of Science and Technology, Gangneung 25451, Korea; jinsoopark@kist.re.kr (J.-S.P.); dekim@snu.ac.kr (D.-E.K.); schong@kist.re.kr (S.-C.H.); hkwon@kist.re.kr (H.C.K.); 2 Department of Pharmaceutical Engineering & Biotechnology, Sunmoon University, Chungnam 31460, Korea; sykim01@sunmoon.ac.kr; 3 Department of Chemistry and Cosmetics, Jeju National University, Jeju 63243, Korea; 4 Smart Farm Research Center, Korea Institute of Science and Technology, Gangneung 25451, Korea

**Keywords:** *Streptomyces nojiriensis* JCM 3382, antibiotics, nojirimycin, biosynthetic gene cluster, secondary metabolism, comparative genomics

## Abstract

*Streptomyces* spp. have been major contributors of novel natural products that are used in many application areas. We found that the nojirimycin (NJ) producer JCM 3382 has antimicrobial activity against *Staphylococcus aureus* via cellular degradation. Genome analysis revealed 30 biosynthetic gene clusters, including those responsible for producing antibiotics, including an azasugar NJ. In-depth MS/MS analysis confirmed the production of 1-deoxynojirimycin (DNJ) along with NJ. In addition, the production of tambromycins, setomimycin, and linearmycins was verified by spectroscopic analyses, including LC-MS and NMR. The distribution of the clusters of genes coding for antibiotics in 2061 *Streptomyces* genomes suggested potential producers of tambromycin, setomimycin, and linearmycin. For a DNJ gene cluster, homologs of *gabT1* and *gutB1* were commonly found; however, *yktC1* was identified in only 112 genomes. The presence of several types of clusters suggests that different strains may produce different types of azasugars. Chemical-profile-inspired comparative genome analysis may facilitate a more accurate assessment of the biosynthetic potential to produce secondary metabolites.

## 1. Introduction

*Streptomyces*, the largest genus in the phylum Actinobacteria, includes aerobic, gram-positive filamentous bacteria distributed in a wide range of environments, from terrestrial to marine. These bacteria have a complex life cycle, producing substrate hyphae, aerial hyphae, and spores [1]. *Streptomyces* spp. are known to be versatile producers of antibiotics and generate diverse chemical scaffolds using a variety of biochemical mechanisms [2]. These microbe-derived chemical scaffolds have been used directly as drug candidates and have provided structural insights into the design of pharmacophore models [3]. Recent advances in DNA sequencing technology have led to the generation of a large amount of genome sequence data from thousands of *Streptomyces* strains. This large volume of data provides an unprecedented opportunity to explore the genetic basis of the biosynthesis of valuable secondary metabolites produced by these organisms.

For the last two decades, extensive genome mining has been commonly carried out, and the resulting data have aided in the discovery and characterization of natural products. This so-called forward approach has revolutionized methods for linking genes to metabolites. The previously used reverse/retro approach involves understanding biosynthetic pathways from isolated chemical structures [4,5], whereas the forward approach facilitates the investigations of genes, proteins, and pathways in a systems context. Coelicheilin, stambromycin, venemycin, argimycin P, and avermipeptin analogs were discovered in *S. coelicolor* M145, *S. ambofaciens* ATCC 23877, *S. venezulae* ATCC 10712, *S.* *argillaceus* ATCC 12956, and *S. actuosus* ATCC 25421, respectively, using the forward approach [6,7,8,9,10]. Despite such efforts, most biosynthetic gene clusters (BGCs) remain inactive under common culture conditions, hampering the investigation of cryptic and silent clusters. The analysis of complete genomes, coupled with metabolic analysis, will facilitate the linkage of more BGCs with the compounds they produce, especially for strains that are yet to be studied in detail [11].

Here, we report the complete genome of *Streptomyces nojiriensis* JCM 3382, which produces three antibiotics (tambromycins, setomimycin, and linearmycins) and NJ. The strain JCM 3382 has shown antibacterial activity against the pathogenic bacterium *Staphylococcus aureus* ATCC 25923, exerted via cellular degradation. The genes in the BGCs of the strain JCM 3382 were compared with those of a broad range of *Streptomyces* spp. Although the strain JCM 3382 is known to produce NJ, a potent alpha-glucosidase inhibitor [12,13,14], the BGCs and pathways involved in the production of NJ have not been fully characterized in the genus *Streptomyces*. Comparative genome analysis and chemical profiling of the strain JCM 3382 have revealed the importance of their BGCs in azasugar production in the genus *Streptomyces*. The chemical profiling of the strain JCM 3382 and comprehensive in silico analysis could provide new insights into the diversity of antibiotics as well as the biology of azasugar-producing microbes.

## 2. Materials and Methods

### 2.1. Isolation of Genomic DNA and Genome Sequencing

*S. nojiriensis* strain JCM 3382 was obtained from the Japan Collection of Microorganisms and maintained in YM or BFM medium. YM medium consisted of 0.5% yeast extract and 3% malt in distilled water, and BFM medium was made of 1% yeast extract, 0.5% glucose, 0.05% K_2_HPO_4_, 0.05% MgSO_4_·7H_2_O, 0.05% NaCl, and 0.05% KNO_3_ in distilled water. Cells were harvested by centrifugation, and genomic DNA was isolated using the SolGent™ genomic DNA Prep kit according to the manufacturer’s protocol (SolGent, Daejeon, Korea). The genome of the strain JCM 3382 was sequenced by PacBio RSII technology (Macrogen, Inc., Seoul, Korea). The PacBio sequencing generated 169,055 reads, with a total of 1,660,471,125 bases.

### 2.2. Genome Assembly and Annotation

The PacBio reads were assembled using CANU (v1.7) [15]. The completeness of the genome assembly was estimated by analyzing the Actinobacteria dataset (*odb9*) of Benchmarking Universal Single-Copy Orthologs (BUSCO v3.0.2) [16]. Gene prediction for the complete genome sequence was performed using Prokka (v1.13) [17]. RNA genes were predicted using RNAmmer (v1.2) [18]. Genomic features, predicted genes, and the distribution of BGCs were visualized in a circular plot using Circos (v0.69–9) [19]. Functional annotation of the predicted genes was performed using eggNOG-mapper and visualized using the ggplot2 package in R (v4.0.3) [20,21,22].

### 2.3. Spectrometric Experiments

ESIMS spectra were obtained on an Agilent Technologies 1200 series/Agilent 6120 Quadrupole LC/MS instrument (Agilent, Santa Clara, CA, USA) with a Phenomenex Luna^®^ C18 (2) 5 μm (4.6 × 150 mm) column (Phenomenex, Torrance, CA, USA). HRESIMS spectra were obtained using a Q Exactive™ Hybrid Quadrupole-Orbitrap™ mass spectrometer (Thermo Fisher Scientific, Waltham, MA, USA) with Kinetex^®^ 2.6 μm HILIC 100 Å (2.1 × 100 mm) columns (Phenomenex, Torrance, CA, USA). Preparative HPLC was performed using a Luna^®^ C8 (2) 100 Å (10 × 250 mm) 10 μm column (Phenomenex, Torrance, CA, USA). All analytical chromatography was performed using mobile phases that consisted of 0.05% formic acid in water (A) and acetonitrile (B). The HPLC-MS data were obtained with the following gradient method: 0–30 min, 10–100% B; 30–35 min, 100% B. The flow rate was set at 0.7 mL/min. The HR-MS analysis was performed using the following gradient elution method: 0–3 min, 90% B; 3–10 min, 90–50% B; 10–13 min, 50% B. The flow rate was set at 0.3 mL/min. Full MS spectra were acquired in positive-ionization conditions from *m/z* 100 to *m/z* 1500 at 70,000 FWHM resolution, with MS/MS fragmentation data obtained in data-dependent scan mode using 30 V collision energies at 17,500 FWHM resolution. NMR spectra were obtained on a Varian Unity Plus 500 MHz NMR system (Varian Co., Palo Alto, CA, USA), and samples were dissolved in methanol-*d*_4_.

### 2.4. Identification of Secondary Metabolites and Molecular Network Analysis

A 30 mL culture of JCM 3382 was incubated for 3 days at 28 °C in a 100 mL Erlenmeyer flask and subsequently used to inoculate a 1% seed culture of 400 mL YM medium in a 1 L Erlenmeyer flask. This culture was incubated for 7 days at 28 °C on a platform shaker rotating at 200 rpm. Then, 10 mL of the culture was inoculated into each of six 2.8 L Erlenmeyer flasks containing 1 L of YM medium. After 7 days of cultivation, 6 L of YM broth was centrifuged at 12,000 rpm for 20 min. The pellet was extracted with acetone (70%, *v/v*) and filtered through filter paper. The filtrate was concentrated by rotary evaporation (12.67 g). The concentrate was used for LC-MS analysis with an in-house UV spectra database. Five grams of concentrate was fractionated with a polymeric absorbent Amberlite XAD7-HP (Sigma-Aldrich, St. Louis, MO, USA) to yield a tambromycin-rich fraction, and 500 mg of concentrate was fractionated by 2 g C18 SPE eluted with 20 mL of 0.5% NH_4_OH in water and 100 mL of 0.5% NH_4_OH in MeOH. The water fraction was further fractionated with *n*-BuOH (15.2 mg). The *n*-BuOH fraction was purified by preparative HPLC to yield linearmycin (t_R_ = 17.5 min, 0.3 mg). The gradient used was as follows: 0 min at 30% solvent B (10 mM ammonium acetate in 90% acetonitrile (MeCN); A = 10 mM ammonium acetate) and then a linear gradient to 50% B over 60 min; the flow rate was 3 mL/min.

The LC-MS data of crude extracts were converted to a Global Natural Products Social Molecular Networking (GNPS) compatible format (.mzXML) using the GNPS vendor conversion utility and were uploaded to the GNPS server using the FTP client WinSCP. Molecular networks were generated on the GNPS analysis platform under filtration by removing all MS/MS fragment ions within ±17 Da of the precursor *m/z*. The precursor ion mass tolerance and MS/MS fragment ion tolerance were set to 2.0 and 0.5 Da, respectively [23]. The networks were further visualized using Cytoscape (v3.8.2) [24].

### 2.5. Comparative analysis of the Strain JCM 3382 with 2061 Streptomyces Genomes

A total of 2061 genome sequences belonging to the genus *Streptomyces* were retrieved from the GenBank genome database (https://www.ncbi.nlm.nih.gov/genome/ accessed on 12 November 2020) (Table S1). A phylogenomic tree was constructed with the whole proteome sequences using the standalone version of CVTree [25]. The K value for CVTree was set to 6 as this value has been suggested to be optimal for a prokaryotic phylogeny [26]. Query sequences of the DNJ gene cluster were obtained from the JCM 3382 genome. Protein sequences of gene clusters for tambromycin, setomimycin, and linearmycin were obtained from the genome sequences of *Streptomyces* sp. NRRL F-4474, *S. aurantiacus* JA 4570, and *Streptomyces* sp. Mg1, respectively [27,28,29] (Table S2). The collected protein sequences and nucleotide sequences spanning each gene cluster were searched against 2062 genome sequences of *Streptomyces* spp., including the strain JCM 3382, using TBLASTN and BLASTN (v2.10.0+), respectively. Sequence homology searches of antibiotics gene clusters were visualized as circular plots using Circos (v0.69-9) [19]. Similarity search results of GabT1, YktC1, and GutB1 and nucleotide sequences containing the three genes are shown with the phylogenomic tree of the 2062 *Streptomyces* genomes using Graphical Phylogenetic Analysis (GraPhlAn v1.1.4) [30]. Protein domains were predicted using InterProScan (v5.33-72.0) [31]. Genomes having *gabT1*, *yktC1*, and *gutB1* orthologs in 10 kb windows were subject to the in silico prediction of BGCs using antiSMASH (v5.1.2) [32]. The predicted BGCs were further analyzed using BiG-SCAPE (v1.1.2) and CORASON workflows [33] with the Minimum Information about a Biosynthetic Gene (MIBiG) database (v2.1) [34] to see whether they formed networks with the BGCs of known compounds. The generated networks were visualized using Cytoscape (v3.8.2) [24].

## 3. Results and Discussion

### 3.1. Summary of the S. nojiriensis JCM 3382 Genome

The draft genome sequence of *S. nojiriensis* JCM 3382 was deposited in the NCBI GenBank database under accession number BMRL00000000 as a part of the Global Catalogue of Microorganisms 10K type strain sequencing project [35]. The draft genome was assembled into 145 scaffolds consisting of 148 contigs, comprising a total of 8,993,303 bp. We obtained a complete genome of the strain JCM 3382, which enabled us to elucidate the full genomic underpinnings.

**Figure 1:**
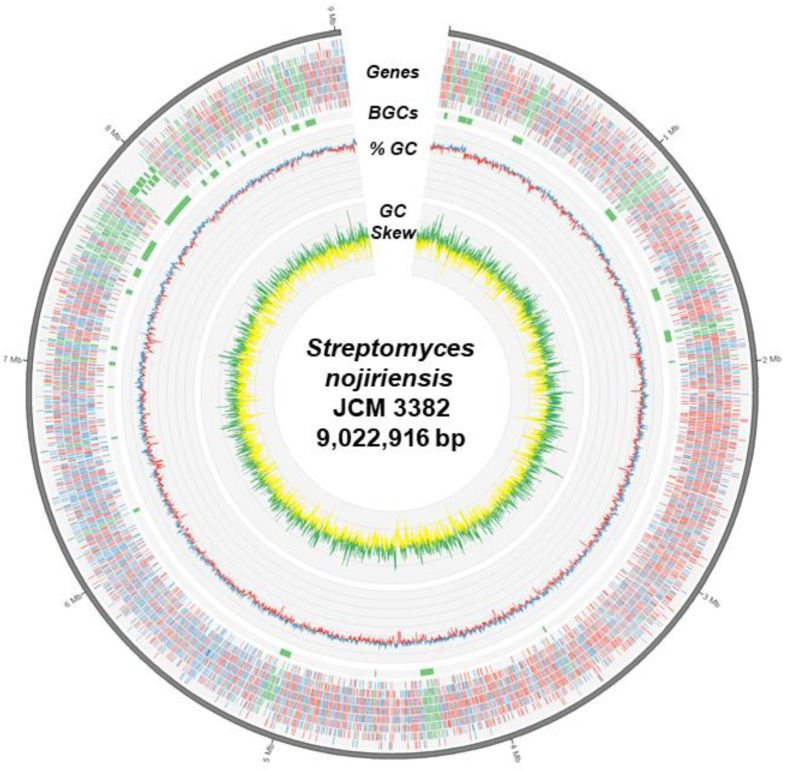
A circular diagram representing the genome of *S. nojiriensis* JCM 3382. From the outermost track to the center: (i) predicted genes (blue/red for forward/reverse strand and green for BGCs), (ii) 30 predicted BGC regions in blocks, (iii) GC content (blue/red for above/below average), and (iv) GC skew (green: >0 and yellow: <0).

The complete genome was assembled into 9,022,916 bp with a GC content of 71.98%. A total of 8103 protein-coding genes were predicted from the genome sequence, including 98.86% of complete BUSCOs (Table 1 and Figure 1). There were 7697 proteins classified into 23 Clusters of Orthologous Groups (COGs) [36]. According to the COG annotations, the five largest groups were the following: unknown function (2417 genes), general function prediction only (1404 genes), transcription (639 genes), amino acid transport and metabolism (388 genes), and signal transduction mechanisms (358 genes) (Figure S1). The genome sequence of the strain JCM 3382 was also predicted to contain 30 BGCs (Table 2). The regions spanning the 30 BGCs included a total of 955 predicted genes, suggesting that the secondary metabolism of this strain may produce a variety of potentially valuable metabolites (Figure 1).

### 3.2. Genome-Mining-Based Identification of Secondary Metabolites Produced by the Strain JCM 3382

To evaluate the potential of JCM 3382 to produce bioactive secondary metabolites, the genome sequence was analyzed using antiSMASH (v5.1.2). Thirty BGCs were found, including six terpene clusters, seven nonribosomal peptide synthetases (NRPSs), three siderophores, and eight polyketide synthases (PKSs; type I, II, and III) (Table 2). A total of 22 clusters exhibited high similarity to known BGCs, whereas the rest were orphan BGCs for which no known homologous clusters could be identified. *Streptomyces* genomes have often been sequenced as fragmented and incomplete assemblies, preventing the identification of BGCs with long, modular proteins consisting of many repeated domains, such as type I PKS and NRPS. For example, only part of the multifunctional megasynthase PKS gene cluster predicted to produce ECO-02301 was detected in the draft genome of JCM 3382. However, in the complete genome, the cluster was fully captured, spanning over 191 kb, including regulatory and accessory genes.

**Figure 2:**
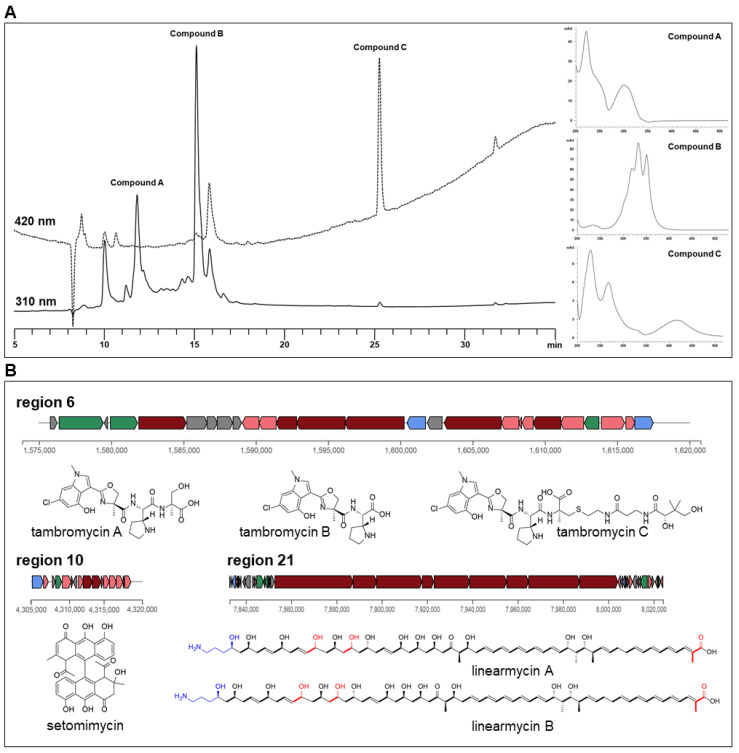
HPLC chromatogram at 310 and 420 nm of JCM 3382 culture extract and metabolites with representative UV absorption spectra (**A**). Biosynthetic gene clusters (regions 6, 10, and 21) activated in this study are shown with their products (**B**).

Although *Streptomyces* strains often possess multiple BGCs, most clusters are cryptic and silent under normal laboratory fermentation conditions. It is therefore important to cultivate these organisms under conditions that facilitate the production of the desired metabolites. We first tried to identify bioactive compounds, other than NJ, that have previously been reported [12]. We observed three classes of metabolites in the HPLC-MS analysis of the culture extract, with significant UV absorption spectra, showing UV maxima at 302 (compound A), 332 (compound B), and 425 nm (compound C), respectively (Figure 2). Our in-house UV library identified the three metabolites as the pyrrolidine-containing peptide JBIR-34, polyene macrolide strevertene, and bianthryl setomimycin, respectively (Figure S2). In the case of compound A, the 3:1 isotope ratio between the [M + H]^+^ and [M + H + 2]^+^ pseudomolecular ion peaks in the MS clearly indicated that the molecule contained one chlorine atom, as observed for JBIR-34 [37]. Its molecular weight of 535 Da indicated that compound A was tambromycin A, which possesses a unique pyrroline-containing amino acid and α-methyl serine instead of the alanine and serine moieties of JBIR-34. Among the expected products of the BGC regions, ECO-02301 had a polyene moiety consisting of five conjugated double bonds, as in strevertene, as its distinct UV absorption spectrum. This UV and MS information enabled each metabolite to be linked to the products of the BGC regions 6, 21, and 10 (Table 2), and this result was further confirmed by HR-MS data, with an identical molecular formula of the two compounds A and C (tambromycin A with *m/z* 536.1907 for [M + H]^+^ and setomimycin with *m/z* 581.1801 for [M + H]^+^) [38,39]. This conclusion was convincingly supported by the additional tandem mass spectra of *m/z* 536.1907, which exactly matched that of tambromycin A [27] (Figure S3). Through a more comprehensive LC-MS exploration of the culture extract, minor metabolites corresponding to tambromycins B and C were also found in the molecular formulas of C_20_H_23_O_5_N_4_Cl and C_35_H_50_O_10_N_7_ClS, respectively, obtained with a high-resolution mass spectrometer.

Compound B had a molecular weight (*m/z* 1118 for [M + H]^+^) different from that of ECO-02301, suggesting that it might be a metabolite with a chromophore similar to that of ECO-02301. A more detailed investigation of PKS in region 21 revealed the presence of three additional extension domains of two malonates and a methylmalonate and the absence of genes for the aminohydroxycyclopentenone moiety and glycosyltransferase compared to the BGC of ECO-02301. The HR-MS result suggested a molecular formula for metabolite B of C_64_H_101_NO_16_ (calc 1140.5030, obsd 1140.7202 for [M + Na]^+^), in accordance with linearmycin A, possessing three additional extending units without any sugar unit, unlike ECO-02301 [29,40]. ^1^H NMR and MS/MS spectra obtained from small quantities also supported the structure (Figure S4). In a further LC-MS analysis, another linearmycin derivative was observed with the molecular weight of linearmycin B, produced by the same biosynthetic machinery in region 21. While linearmycin B was colinearly matched to the number and order of extending modules of modular type I PKS, linearmycin A was produced variably by the module-skipping of the two-carbon unit from malonate elongation prior to the final incorporation of methylmalonyl CoA. Taken together, the information about the molecular formula, UV spectrum, and genome-based analysis led to the identification of the three metabolite groups as tambromycins, linearmycins, and setomimycin. The results implied that the BGCs in regions 6, 10, and 21 were activated under our culture conditions.

Additional molecular networking analysis using the GNPS platform revealed the presence of diverse additional small molecules in the crude extracts of JCM 3382. Molecular networks, including tambromycin and linearmycin, suggested the existence of unknown derivatives (Figure S5).

**Figure 3:**
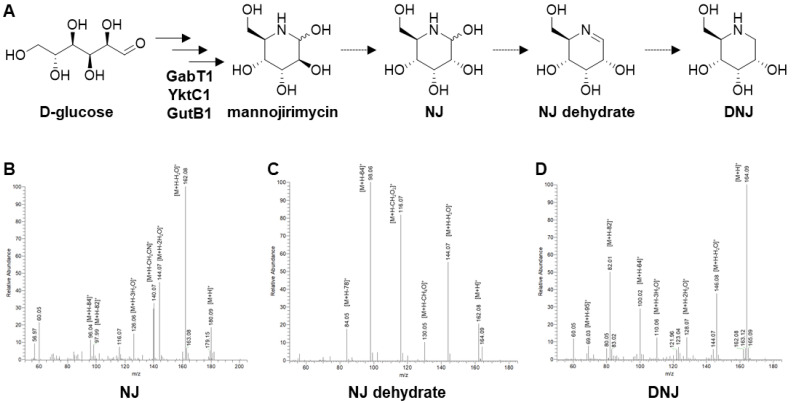
Biosynthetic pathways of DNJ and DNJ intermediates identified using MS/MS. DNJ biosynthetic pathway (**A**) and product ion mass spectra in positive ion mode of molecular ions corresponding to NJ (**B**), NJ dehydrate (**C**), and DNJ (**D**) from the culture extract.

*S. nojiriensis* JCM 3382 was originally reported to be a producer of NJ, and previous studies have suggested that DNJ, a potent inhibitor of alpha-glucosidase, is produced via NJ from glucose [41,42]. More recent studies have reported that the biosynthesis of DNJ is initiated by GabT1, YktC1, and GutB1, which catalyze transamination, dephosphorylation, and oxidoreduction in *Bacillus subtilis* MORI 3K-85, *B. amyloliquefaciens* 140N, and *B. velezensis* K26 [43,44,45] (Figure 3). It has also been reported that NJ and DNJ are produced by a few *Streptomyces* spp. other than the strain JCM 3382 [41,46,47]. A gene cluster containing orthologs of *gabT1*, *yktC1*, and *gutB1* was found in region 20, suggesting the potential for the production of DNJ, in addition to NJ, in this strain (Table 2). It is difficult to isolate and purify azasugars such as NJ and DNJ from culture extract, owing to their high polarity and the absence of a chromophore. Recently, the biosynthetic pathway of DNJ was revealed using tandem MS data, as was the presence of biosynthetic intermediates, including NJ dehydrate, NJ, and 2-amino-2-deoxy-D-mannitol (ADM) [46]. DNJ was identified from the culture extract of the strain JCM 3382 using LC-MS/MS with a HILIC column. The MS/MS spectrum corresponding to DNJ was measured in the culture extract along with the spectra of NJ and NJ dehydrate, as previously reported [46]. The mass ion intensity of DNJ and NJ (1.76e^7^ and 9.12e^5^) on a mass spectrometer also implied that DNJ might be the final product synthesized, with NJ as an intermediate (Figure 3).

### 3.3. Distribution of Gene Clusters for Antibiotics across Streptomyces Genomes

Three antibiotics (tambromycins, setomimycin, and linearmycins) were identified in the strain JCM 3382 using chemical profiling analysis (Figure 2). Only a few strains have been reported to produce these antibiotics: for example, *S. lavendulae* subsp. *lavendulae* NRRL WC-3542 for tambromycin, *S. aurantiacus* JA 4570 and *S. pseudovenezuelae* AM-2947 for setomimycin, and *Streptomyces* sp. Mg1 for linearmycin [27,39,40]. The nucleotide sequences of the whole clusters and the protein sequences produced by each gene cluster were searched against the 2062 *Streptomyces* genomes, including those of the strain JCM 3382, in order to assess the biosynthetic potential of these antibiotics (Tables S3-S5).

**Figure 4:**
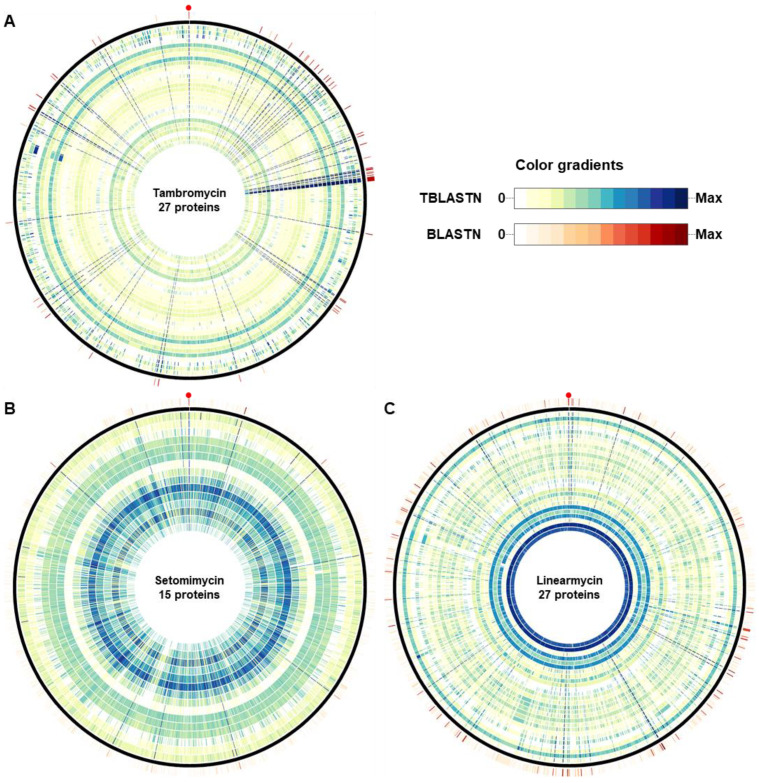
Distribution of proteins in gene clusters producing tambromycin (**A**), setomimycin (**B**), and linearmycin (**C**) in 2062 *Streptomyces* genome sequences. Protein sequences from each cluster were searched using TBLATN with an E-value cutoff of 1e−5. The dark navy-blue color shows the maximum bit score for each protein. The track outside the ideogram shows the sum of bit scores from BLASTN searches when the nucleotide sequences of each cluster were used as the query, with an E-value cutoff of 1e−20. The darker the color of a cell, the more homologous the corresponding sequence is. The strain JCM 3382 is marked by a red dot in each diagram.

Tambromycin is a nonribosomal tetrapeptide that has attracted attention because of its antiproliferative activity in cancerous human B- and T-cell lines [27]. The reference gene cluster was identified in *Streptomyces* sp. NRRL F-4474 and deposited in the MIBiG database [34] under the accession number BGC0001368. The cluster is composed of 27 genes, spanning 42,431 bp (Table S2). The distribution of the proteins produced by the 27 genes clearly highlighted the genomes containing significant hits (Figure 4A). These genomes also showed high bit scores in BLASTN searches, indicating that the order of genes encoding these proteins was collinear to that of the reference cluster (Figure 4A and Table S3). There were 36 genomes containing all the 27 genes clustered within 42,499 bp, on average (Table S6). An ortholog of a gene encoding a 4′-phosphopantetheinyl transferase domain-containing protein (IF33_RS36805) was missing in the predicted gene clusters of 10 genomes, including the strain JCM 3382. For the 10 genomes, 26 genes were clustered within 42,107 bp, on average (Table S6). Since we identified tambromycin from the strain JCM 3382, the 26-gene cluster might be capable of producing tambromycin. This result suggests that these 45 *Streptomyces* spp., other than JCM 3382, may have the metabolic potential to produce tambromycin (Table S6).

Setomimycin, originally discovered in *S. pseudovenezuelae*, exhibits antimicrobial activity against Gram-positive bacteria and antitumor activity [39]. The reference cluster was identified in *S. aurantiacus* JA 4570 and deposited in the MIBiG database [34] under the accession number BGC0002000. The reference cluster consisted of 15 genes, spanning 12,502 bp (Table S2). In contrast to the tambromycin cluster, the protein sequences of the 15 genes showed intricate patterns across the 2062 genomes (Figure 4B). TBLASTN hits of the STRAU_RS10370-encoding gene were found in 613 *Streptomyces* genomes (E-value <1e−5). Interestingly, 11 genomes, including that of the strain JCM 3382, had highly significant hits, with E-values less than 1e−55 (Table S4). The protein STRAU_RS10370 was predicted to belong to the nuclear transport factor 2-like (NTF2-like) superfamily, which is functionally diverse. It includes SnoaL, Lsd19, and SDH1, which catalyze intramolecular aldol condensation, epoxide-opening cyclization, and dehydration, respectively [48,49,50]. Setomimycin is produced from an oxidative coupling between two nonaketidic precursors, similar to the other dimeric pre-anthraquinones julichromes and spectiomycin B1 [28]. However, setomimycin requires dehydration for its anthraquinone unit, unlike other compounds. An NTF2-like protein (STRAU_RS10370) is only found in the setomimycin BGC; thus, the enzyme might function as a dehydratase in the biosynthetic pathway. These 11 genomes contained hits to all the 15 proteins and showed high similarity in BLASTN results, suggesting that they may have the genetic potential to produce setomimycin (Figure 4B and Table S6).

Linearmycin is a linear polyene antibiotic with antifungal activity [40]. The genome sequence of *Streptomyces* sp. Mg1 contained a cluster of 27 genes, including 9 polyketide synthase genes (Supplementary Table S2) [29]. Only six genomes had highly significant hits to the 27 genes clustered within 175,401 bp, on average (Figure 4C and Table S6). Four enzymes (LnyI, LnyN, LnyO, and LnyT) are responsible for producing the aminoalkyl starter unit from arginine. Linearmycins are known to be released from acyl carrier proteins by a terminal thioesterase at PKS (LnyHI) to yield a linear polyketide product [29,51]. Collectively, these five proteins were selected as key enzymes for the linearmycin cluster. Sequence similarity searches showed that the six genomes had highly significant hits to these key enzymes (Table S5). There were 13 additional genomes that had significant hits to the 27 genes, but these were located in multiple scaffolds and/or scattered at distant locations. Further efforts to obtain complete genomes would enable accurate genomic assessment.

In addition to JCM 3382, based on the nucleotide similarity to the reference clusters and the presence of genes in the clusters, 45 *Streptomyces* genomes were predicted to have the genetic machinery necessary for producing tambromycin, 10 were predicted to produce setomimycin, and 5 to produce linearmycin. Further investigations involving metabolite analyses and molecular studies would validate their ability to produce antibiotics and also facilitate more accurate prediction based on genome mining.

### 3.4. Distribution of gabT1, yktC1, and gutB1 across 2062 Streptomyces Genomes

**Figure 5:**
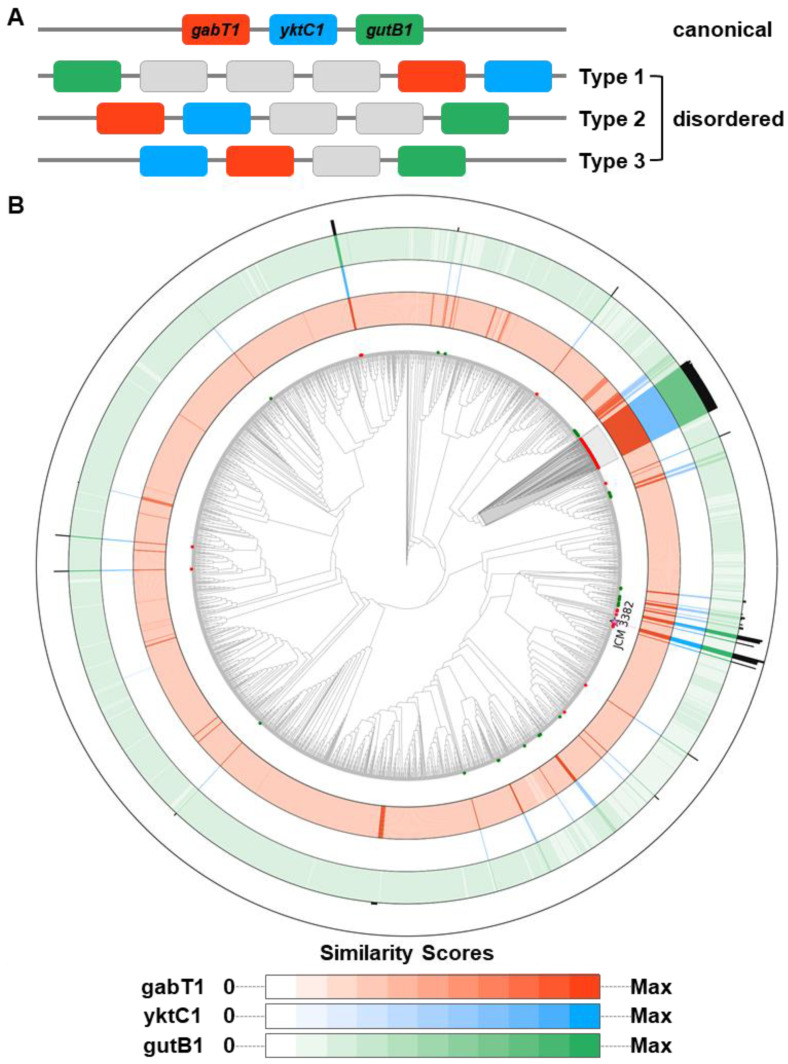
Distribution of the *gabT1*-*yktC1*-*gutB1* gene cluster over a phylogenomic tree of 2062 *Streptomyces* genomes. (**A**) Schematic diagrams describing the gene structure. *gabT1*, *yktC1*, and *gutB1* are indicated by red, blue, and green, respectively. Gray indicates a gene other than these three. (**B**) Distribution of the gene cluster with the phylogenomic tree shown as a cladogram. A total of 110 genomes are indicated by colors in their terminal nodes: genomes containing (i) a canonical gene cluster (red), (ii) a canonical and a disordered cluster (pink), and (iii) a disordered cluster (green). The strain JCM 3382 is indicated with a star at the terminal node. A clade shaded in gray indicates 53 closely related genomes having a canonical cluster. The sequence homologies of GabT1, YktC1, and GutB1 are shown in color gradients. The more intense the color, the more homologous genes found in the corresponding genome. Homology was measured using the formula −log_10_ (E-value). An E-value of 0 was replaced with 1e−200 to avoid infinity. The homology of the nucleotide sequences of the whole gene cluster was calculated as the sum of significant hits (E-value <1e−20) and is shown as a histogram. From the outermost track: the whole gene cluster, GutB1, YktC1, and GabT1.

It has been reported that three proteins, GabT1, YktC1, and GutB1, are responsible for the initiation of DNJ biosynthesis [42,43,44,45]. To date, only a few species in the genera *Bacillus* and *Streptomyces* have been reported to produce DNJ [45]. The distribution of these three genes in *Bacillus* spp. was recently investigated [45] but has not yet been investigated in *Streptomyces* spp. A total of 2062 genomes in the genus *Streptomyces*, including that of the strain JCM 3382, were investigated for their potential for DNJ biosynthesis (Table S1). The distribution of the three protein sequences varied greatly across the 2062 *Streptomyces* genomes. Only a few strains had homologous genes encoding the three proteins with strong identity, while other strains only had remotely homologous genes encoding GabT1 and GutB1 (Figure 5 and Table S7). Homologous hits for GabT1 and GutB1 were detected in all genomes analyzed; however, significant hits for YktC1 were found only in 112 genomes (E-value <1e−88) (Table S7). In general, orthologs of GabT1 and GutB1 were a great deal more significant when a YktC1 hit was present than in the absence of a YktC1 ortholog (Table S7).

In order to identify putative DNJ gene clusters, genomic regions harboring *gabT1*, *yktC1*, and *gutB1* within a 10 kb window were retrieved. As a result, 114 clusters in 110 genomes were identified from 2062 *Streptomyces* genomes (Table S8). There were 73 genomes with a “canonical” cluster, with the order *gabT1*-*yktC1*-*gutB1* spanning 3251 bp, on average (Figure 5 and Table S8). Interestingly, 53 out of the 73 genomes formed a clade on a phylogenomic tree of the 2062 genomes (Figure 5; shaded in grey). There were 41 genomes having orthologs of the three genes but with a more “disordered” structure than the canonical clusters. In the disordered clusters, *gabT1*, *yktC1*, and *gutB1* were interleaved with one to three additional genes. There were 98 proteins encoded by the interleaved genes, which could be classified into seven groups based on domain profiles. A majority of the proteins, 88 out of 98, belonged to four groups predicted to have (i) an aminoglycoside phosphotransferase domain (IPR002575) and a protein kinase-like domain (IPR011009); (ii) an ROK (repressor, ORF, kinase) family domain (IPR000600) and an ATPase nucleotide-binding domain (IPR043129); (iii) intradiol ring-cleavage dioxygenase domains (IPR000627 and IPR015889); and (iv) sugar nucleotide and NAD(P)-binding domains (IPR029903 and IPR036291) (Figure S6). Strains JCM 3382, *S. subrutilus* ATCC 27467, and *S. subrutilus* JCM 4834 had a disordered cluster as well as a canonical one at distant chromosomal locations (Table S8).

BGCs were predicted for the 110 genomes harboring *gabT1*, *yktC1*, and *gutB1* in 10 kb and further analyzed by the BiG-SCAPE/CORASON workflow [33]. As a result, 58 out of 114 *gabT1*-*yktC1*-*gutB1*-containing BGCs were predicted by antiSMASH, and 42 out of the 58 BGCs were found in the resulting networks (Figure S7). The 42 BGCs did not form a network with those from the MIBiG database. They formed 12, mostly isolated, networks. In particular, nine BGCs having the disordered Type 2 and six BGCs having the disordered Type 3 *gabT1*-*yktC1*-*gutB1* clusters formed a single network by themselves, respectively (Figure S7). This organization might suggest that these BGCs have a unique metabolic potential for producing different types of azasugar. JCM 3382, a BGC containing a canonical *gabT1*-*yktC1*-*gutB1* cluster, was included in the network, but the other disordered *gabT1*-*yktC1*-*gutB1* cluster was not predicted by antiSMASH. This result may imply that some of the disordered Type 1 gene sets undergo functional diversification and produce metabolites other than DNJ and DNJ-like azasugars.

Orthologs of the three genes have also been found in other bacterial species. *Mycobacteroides abscessus* subsp. *massiliense* carries SLB54085.1 (an ortholog of GabT1), SLB54050.1 (an ortholog of YktC1), and SLB54030.1 (an ortholog of GutB1). A cluster of eight genes in *Paenibacillus polymyxa* SC2 has the potential to produce DNJ biosynthesis, initiated by a pyridoxal 5′-phosphate-dependent transamination by a GabT1 ortholog (ADO56555.1) [52]. In the proposed cluster, an ortholog of *glcP1* encoding a putative fucose permease (ADO56556.1) was located close to the *gabT1* ortholog. The protein sequence of GlcP1 was searched against the 2062 *Streptomyces* genomes. A total of 363 poor hits, with sequence identities between 20.85% and 27.86%, was identified using BLASTP searches, implying that the proposed cluster in *P*. *polymyxa* may not be conserved in the genus *Streptomyces*. In *Chitinophaga pinensis* DSM 2588, orthologs of GabT1 and GutB1 are involved in the biosynthesis of another azasugar, 1,4-dideoxy-1,4-aminoarabinitol (DAB-1) [53].

In summary, 73 genomes with the canonical *gabT1*-*yktC1*-*gutB1* cluster may have the genetic potential to catalyze the initial steps of DNJ biosynthesis. Considering that a significant ortholog of *glcP1* was not found in the 2062 *Streptomyces* genomes, there might be multiple pathways of DNJ biosynthesis. Disordered clusters may be subject to neofunctionalization and may, therefore, have the potential to produce DNJ derivatives or different types of azasugars. Further metabolic and chemical studies are needed to validate the biosynthetic potential of these clusters.

## 4. Conclusions

Chemical profiling using spectrometric methods has revealed that the strain JCM 3382 produces three antibiotics along with azasugars, as predicted by the genome-mining of BGCs. Comparative genome analysis identified putative antibiotic-producing strains with the potential to catalyze the initial steps of DNJ biosynthesis. Analysis of the DNJ gene cluster suggested that the three genes might have undergone long-standing evolution for metabolic diversity. As presented in this study, chemical-profile-inspired comparative genome analysis may facilitate the more accurate assessment of biosynthetic potential.

## Figures and Tables

**Table 1:** Genomic features of *S. nojiriensis* JCM 3382.

Genomic Feature	Value
Size of the genome assembly (bp)	9,022,916
GC content (%)	71.98
Protein-coding genes/regions (bp)	8103/7,869,321
tRNA/tmRNA/rRNA genes	99/1/21
Genes assigned to COG categories	7697
Complete BUSCOs (%)	98.86

**Table 2:** Predicted biosynthetic gene clusters of *S. nojiriensis* JCM 3382.

Region	Type	Most Similar Known Cluster	Similarity
Region 1	Terpene	Avermitilol	100.00%
Region 2	Lanthipeptide, PKS-like, Butyrolactone	Venezuelin	100.00%
Region 3	Thiopeptide, LAP	Lactazole	55.00%
Region 4	NRPS	Deimino-antipain	66.00%
Region 5	T2PKS	Spore pigment	58.00%
Region 6	NRPS	JBIR-126 (tambromycin)	96.00%
Region 7	NRPS, T1PKS	Coelichelin	72.00%
Region 8	Butyrolactone	Neocarzinostatin	4.00%
Region 9	Siderophore	Desferrioxamin B	100.00%
Region 10	T2PKS	Setomimycin	100.00%
Region 11	RiPP-like		
Region 12	NRPS, T1PKS	Bleomycin	9.00%
Region 13	CDPS	BD-12	17.00%
Region 14	Siderophore	Ficellomycin	3.00%
Region 15	Bacteriocin		
Region 16	Terpene	Toxoflavin, Fervenulin	14.00%
Region 17	Lanthipeptide		
Region 18	Terpene	Hopene	61.00%
Region 19	T1PKS, hglE-KS		
Region 20	NRPS, NRPS-like	Kirromycin, Nojirimycin	22.00%
Region 21	T1PKS	ECO-02301	75.00%
Region 22	Lanthipeptide		
Region 23	NRPS-like	Lipstatin	42.00%
Region 24	Terpene		
Region 25	Terpene	Ebelactone	5.00%
Region 26	Terpene	Monensin	5.00%
Region 27	Melanin	Istamycin	4.00%
Region 28	Siderophore		
Region 29	T3PKS	Alkylresorcinol	100.00%
Region 30	CDPS, NRPS-like		

## Data Availability

The complete genome sequence of *Streptomyces nojiriensis* strain JCM 3382 was deposited in the NCBI GenBank under accession number CP071139 (BioProject: PRJNA704480). Mass spectrometry data were deposited with the Mass Spectrometry Interactive Virtual Environment (dataset identifier: MSV000087924), which can be accessed from https://doi.org/10.25345/C5MZ61 (accessed on 19 August 2021). Network data from Figures S5 and S7 are included in the Supplementary Materials as Cytoscape session files (*.cys).

## Supplementary Materials

The following are available online at https://www.mdpi.com/article/10.3390/microorganisms9091802/s1, Figure S1: COG functional annotation of the predicted proteins in the strain JCM 3382. Figure S2: In-house UV library screening results for three identified molecules from a culture extract of JCM 3382. Figure S3: Tandem mass spectrum of tambromycin A in the positive mode, and the assignment of fragment ions. Figure S4: MS/MS and ^1^H NMR data of linearmycin A isolated from JCM 3382. Figure S5: Molecular networks constructed with LC-MS/MS data from the culture extract produced by JCM 3382. Figure S6: Distribution of protein sequences encoded by 98 interleaved genes in disordered clusters by domain profiles. Figure S7: Visualization of BGC networks generated by BiG-SCAPE. Table S1: List of 2061 *Streptomyces* genomes used in the comparative analysis. Table S2: Reference gene clusters of tambromycin, setomimycin, and linearmycin. Table S3: TBLASTN and BLASTN results of the 27 proteins and the whole nucleotide sequences of the tambromycin gene cluster in the 2062 *Streptomyces* genomes. Table S4: TBLASTN and BLASTN results of the 15 proteins and the whole nucleotide sequences of the setomimycin gene cluster in the 2062 *Streptomyces* genomes. Table S5: TBLASTN and BLASTN results of the 27 proteins and the whole nucleotide sequences of the linearmycin gene cluster in the 2062 *Streptomyces* genomes. Table S6: List of putative antibiotic gene clusters found in 2062 *Streptomyces* genomes. Table S7: TBLASTN and BLASTN results of the three proteins and the whole nucleotide sequences of the putative *gabT1*-*yktC1*-*gutB1* gene cluster in the 2062 *Streptomyces* genomes. Table S8: List of 114 putative *gabT1*-*yktC1*-*gutB1* gene clusters found in 2062 *Streptomyces* genomes.

## References

[1] Claessen D., de Jong W., Dijkhuizen L., Wosten H.A. (2006). Regulation of *Streptomyces* development: Reach for the sky!. Trends Microbiol..

[2] van Bergeijk D.A., Terlouw B.R., Medema M.H., van Wezel G.P. (2020). Ecology and genomics of Actinobacteria: New concepts for natural product discovery. Nat. Rev. Microbiol..

[3] Katz L., Baltz R.H. (2016). Natural product discovery: Past, present, and future. J. Ind. Microbiol. Biotechnol..

[4] Ziemert N., Alanjary M., Weber T. (2016). The evolution of genome mining in microbes—A review. Nat. Prod. Rep..

[5] Khater S., Anand S., Mohanty D. (2016). In silico methods for linking genes and secondary metabolites: The way forward. Synth. Syst. Biotechnol..

[6] Laureti L., Song L.J., Huang S., Corre C., Leblond P., Challis G.L., Aigle B. (2011). Identification of a bioactive 51-membered macrolide complex by activation of a silent polyketide synthase in *Streptomyces ambofaciens*. Proc. Natl. Acad. Sci. USA.

[7] Thanapipatsiri A., Gomez-Escribano J.P., Song L.J., Bibb M.J., Al-Bassam M., Chandra G., Thamchaipenet A., Challis G.L., Bibb M.J. (2016). Discovery of unusual biaryl polyketides by activation of a silent *Streptomyces venezuelae* biosynthetic gene cluster. ChemBioChem.

[8] Ye S.H., Molloy B., Brana A.F., Zabala D., Olano C., Cortes J., Moris F., Salas J.A., Mendez C. (2017). Identification by genome mining of a type I polyketide gene cluster from *Streptomyces argillaceus* involved in the biosynthesis of pyridine and piperidine alkaloids argimycins P. Front. Microbiol..

[9] Lautru S., Deeth R.J., Bailey L.M., Challis G.L. (2005). Discovery of a new peptide natural product by *Streptomyces coelicolor* genome mining. Nat. Chem. Biol..

[10] Liu W.Y., Sun F.X., Hu Y. (2018). Genome mining-mediated discovery of a new avermipeptin analogue in *Streptomyces actuosus* ATCC 25421. ChemistryOpen.

[11] Baltz R.H. (2011). Strain improvement in actinomycetes in the postgenomic era. J. Ind. Microbiol. Biotechnol..

[12] Ishida N., Kumagai K., Niida T., Hamamoto K., Shomura T. (1967). Nojirimycin, a new antibiotic. I. Taxonomy and fermentation. J. Antibiot. (Tokyo).

[13] Ishida N., Kumagai K., Niida T., Tsuruoka T., Yumoto H. (1967). Nojirimycin, a new antibiotic. II. Isolation, characterization and biological activity. J. Antibiot. (Tokyo).

[14] Niwa T., Inouye S., Tsuruoka T., Koaze Y., Niida T. (1970). “Nojirimycin” as a potent inhibitor of glucosidase. Agric. Biol. Chem..

[15] Koren S., Walenz B.P., Berlin K., Miller J.R., Bergman N.H., Phillippy A.M. (2017). Canu: Scalable and accurate long-read assembly via adaptive *k*-mer weighting and repeat separation. Genome Res..

[16] Simao F.A., Waterhouse R.M., Ioannidis P., Kriventseva E.V., Zdobnov E.M. (2015). BUSCO: Assessing genome assembly and annotation completeness with single-copy orthologs. Bioinformatics.

[17] Seemann T. (2014). Prokka: Rapid prokaryotic genome annotation. Bioinformatics.

[18] Lagesen K., Hallin P., Rodland E.A., Staerfeldt H.H., Rognes T., Ussery D.W. (2007). RNAmmer: Consistent and rapid annotation of ribosomal RNA genes. Nucleic Acids Res..

[19] Krzywinski M., Schein J., Birol I., Connors J., Gascoyne R., Horsman D., Jones S.J., Marra M.A. (2009). Circos: An information aesthetic for comparative genomics. Genome Res..

[20] Huerta-Cepas J., Szklarczyk D., Heller D., Hernandez-Plaza A., Forslund S.K., Cook H., Mende D.R., Letunic I., Rattei T., Jensen L.J. (2019). eggNOG 5.0: A hierarchical, functionally and phylogenetically annotated orthology resource based on 5090 organisms and 2502 viruses. Nucleic Acids Res..

[21] Wickham H. (2009). ggplot2: Elegant Graphics for Data Analysis.

[22] R Core Team (2020). R: A Language and Environment for Statistical Computing.

[23] Wang M., Carver J.J., Phelan V.V., Sanchez L.M., Garg N., Peng Y., Nguyen D.D., Watrous J., Kapono C.A., Luzzatto-Knaan T. (2016). Sharing and community curation of mass spectrometry data with global natural products social molecular networking. Nat. Biotechnol..

[24] Shannon P., Markiel A., Ozier O., Baliga N.S., Wang J.T., Ramage D., Amin N., Schwikowski B., Ideker T. (2003). Cytoscape: A software environment for integrated models of biomolecular interaction networks. Genome Res..

[25] Qi J., Wang B., Hao B.I. (2004). Whole proteome prokaryote phylogeny without sequence alignment: A *K*-string composition approach. J. Mol. Evol..

[26] Zuo G., Xu Z., Yu H., Hao B. (2010). Jackknife and bootstrap tests of the composition vector trees. Genom. Proteom. Bioinform..

[27] Goering A.W., McClure R.A., Doroghazi J.R., Albright J.C., Haverland N.A., Zhang Y., Ju K.S., Thomson R.J., Metcalf W.W., Kelleher N.L. (2016). Metabologenomics: Correlation of microbial gene clusters with metabolites drives discovery of a nonribosomal peptide with an unusual amino acid monomer. ACS Cent. Sci..

[28] Prag A., Gruning B.A., Hackh M., Ludeke S., Wilde M., Luzhetskyy A., Richter M., Luzhetska M., Gunther S., Muller M. (2014). Regio- and stereoselective intermolecular oxidative phenol coupling in *Streptomyces*. J. Am. Chem. Soc..

[29] Hoefler B.C., Stubbendieck R.M., Josyula N.K., Moisan S.M., Schulze E.M., Straight P.D. (2017). A link between linearmycin biosynthesis and extracellular vesicle genesis connects specialized metabolism and bacterial membrane physiology. Cell Chem. Biol..

[30] Asnicar F., Weingart G., Tickle T.L., Huttenhower C., Segata N. (2015). Compact graphical representation of phylogenetic data and metadata with GraPhlAn. PeerJ.

[31] Blum M., Chang H.Y., Chuguransky S., Grego T., Kandasaamy S., Mitchell A., Nuka G., Paysan-Lafosse T., Qureshi M., Raj S. (2021). The InterPro protein families and domains database: 20 years on. Nucleic Acids Res..

[32] Blin K., Shaw S., Steinke K., Villebro R., Ziemert N., Lee S.Y., Medema M.H., Weber T. (2019). antiSMASH 5.0: Updates to the secondary metabolite genome mining pipeline. Nucleic Acids Res..

[33] Navarro-Munoz J.C., Selem-Mojica N., Mullowney M.W., Kautsar S.A., Tryon J.H., Parkinson E.I., De Los Santos E.L.C., Yeong M., Cruz-Morales P., Abubucker S. (2020). A computational framework to explore large-scale biosynthetic diversity. Nat. Chem. Biol..

[34] Kautsar S.A., Blin K., Shaw S., Navarro-Munoz J.C., Terlouw B.R., van der Hooft J.J.J., van Santen J.A., Tracanna V., Suarez Duran H.G., Pascal Andreu V. (2020). MIBiG 2.0: A repository for biosynthetic gene clusters of known function. Nucleic Acids Res..

[35] Wu L., Ma J. (2019). The Global Catalogue of Microorganisms (GCM) 10K type strain sequencing project: Providing services to taxonomists for standard genome sequencing and annotation. Int. J. Syst. Evol. Microbiol..

[36] Galperin M.Y., Wolf Y.I., Makarova K.S., Vera Alvarez R., Landsman D., Koonin E.V. (2021). COG database update: Focus on microbial diversity, model organisms, and widespread pathogens. Nucleic Acids Res..

[37] Motohashi K., Takagi M., Shin-Ya K. (2010). Tetrapeptides possessing a unique skeleton, JBIR-34 and JBIR-35, isolated from a sponge-derived actinomycete, *Streptomyces* sp. Sp080513GE-23. J. Nat. Prod..

[38] Izumikawa M., Kawahara T., Kagaya N., Yamamura H., Hayakawa M., Takagi M., Yoshida M., Doi T., Shin-ya K. (2015). Pyrrolidine-containing peptides, JBIR-126,-148, and-149, from *Streptomyces* sp. NBRC 111228. Tetrahedron Lett..

[39] Omura S., Tanaka H., Iwai Y., Nishigaki K., Awaya J., Takahashi Y., Masuma R. (1978). A new antibiotic, setomimycin, produced by a strain of *Streptomyces*. J. Antibiot. (Tokyo).

[40] Sakuda S., Guce-Bigol U., Itoh M., Nishimura T., Yamada Y. (1995). Linearmycin a, a novel linear polyene antibiotic. Tetrahedron Lett..

[41] Hardick D.J., Hutchinson D.W., Trew S.J., Wellington E.M.H. (1991). The biosynthesis of deoxynojirimycin and deoxymannonojirimycin in *Streptomyces subrutilus*. J. Chem. Soc. Chem. Commun..

[42] Hardick D.J., Hutchinson D.W., Trew S.J., Wellington E.M.H. (1992). Glucose is a precursor of 1-deoxynojirimycin and 1-deoxymannonojirimycin in *Streptomyces subrutilus*. Tetrahedron.

[43] Kang K.D., Cho Y.S., Song J.H., Park Y.S., Lee J.Y., Hwang K.Y., Rhee S.K., Chung J.H., Kwon O., Seong S.I. (2011). Identification of the genes involved in 1-deoxynojirimycin synthesis in *Bacillus subtilis* MORI 3K-85. J. Microbiol..

[44] Clark L.F., Johnson J.V., Horenstein N.A. (2011). Identification of a gene cluster that initiates azasugar biosynthesis in *Bacillus amyloliquefaciens*. ChemBioChem.

[45] Lee H., Jung D.H., Seo D.H., Chung W.H., Seo M.J. (2021). Genome analysis of 1-deoxynojirimycin (1-DNJ)-producing *Bacillus velezensis* K26 and distribution of *Bacillus* sp. harboring a 1-DNJ biosynthetic gene cluster. Genomics.

[46] Wu H., Guo Y., Chen L., Chen G.G., Liang Z.Q. (2019). A novel strategy to regulate 1-deoxynojirimycin production based on its biosynthetic pathway in *Streptornyces lavendulae*. Front. Microbiol..

[47] Argoudelis A.D., Reusser F., Mizsak S.A., Baczynskyj L. (1976). Antibiotics produced by *Streptomyces ficellus* II. Feldamycin and nojirimycin. J. Antibiot..

[48] Sultana A., Kallio P., Jansson A., Wang J.S., Niemi J., Mantsala P., Schneider G. (2004). Structure of the polyketide cyclase SnoaL reveals a novel mechanism for enzymatic aldol condensation. EMBO J..

[49] Shichijo Y., Migita A., Oguri H., Watanabe M., Tokiwano T., Watanabe K., Oikawa H. (2008). Epoxide hydrolase Lsd19 for polyether formation in the biosynthesis of lasalocid A: Direct experimental evidence on polyene-polyepoxide hypothesis in polyether biosynthesis. J. Am. Chem. Soc..

[50] Lundqvist T., Rice J., Hodge C.N., Basarab G.S., Pierce J., Lindqvist Y. (1994). Crystal-structure of scytalone dehydratase—A disease determinant of the rice pathogen, *Magnaporthe grisea*. Structure.

[51] McAlpine J.B., Bachmann B.O., Piraee M., Tremblay S., Alarco A.M., Zazopoulos E., Farnet C.M. (2005). Microbial genomics as a guide to drug discovery and structural elucidation: ECO-02301, a novel antifungal agent, as an example. J. Nat. Prod..

[52] Arciola J.M., Horenstein N.A. (2018). Characterization of the PLP-dependent transaminase initiating azasugar biosynthesis. Biochem. J..

[53] Nunez C., Horenstein N.A. (2019). Functional analysis of a gene cluster from *Chitinophaga pinensis* involved in biosynthesis of the pyrrolidine azasugar DAB-1. J. Nat. Prod..

