# Supplementary material for: Genome Analysis of Streptomyces nojiriensis JCM 3382 and Distribution of Gene Clusters for Three Antibiotics and an Azasugar across the Genus Streptomyces
Source: Microorganisms. 2021 Aug 25;9(9):1802. doi: 10.3390/microorganisms9091802 (PMC8466323; doi:10.3390/microorganisms9091802)
Supplement: Supplementary file 1 [file microorganisms-09-01802-s001.zip › Supplementary_Materials.pdf]

Supplementary Materials

Genome analysis of *Streptomyces nojiriensis* JCM 3382 and distribution of gene clusters for three antibiotics and an azasugar across the genus *Streptomyces*

Jin-Soo Park[1], Da-Eun Kim[1], Sung-Chul Hong[1], Seung-Young Kim[2], Hak Cheol Kwon[1], Chang-Gu Hyun[3,*] and Jaeyoung Choi[4,*]

[1] Natural Product Informatics Research Center, Korea Institute of Science and Technology, Gangneung 25451, Republic of Korea

[2] Department of Pharmaceutical Engineering & Biotechnology, Sunmoon University, Chungnam 31460, Republic of Korea

[3] Department of Chemistry and Cosmetics, Jeju National University, Jeju 63243, Republic of Korea

[4] Smart Farm Research Center, Korea Institute of Science and Technology, Gangneung 25451, Republic of Korea

* Corresponding authors:

E-mail addresses: cghyun@jejunu.ac.kr (C.-G.H.), jaeyoung.choi@kist.re.kr (J.C.)

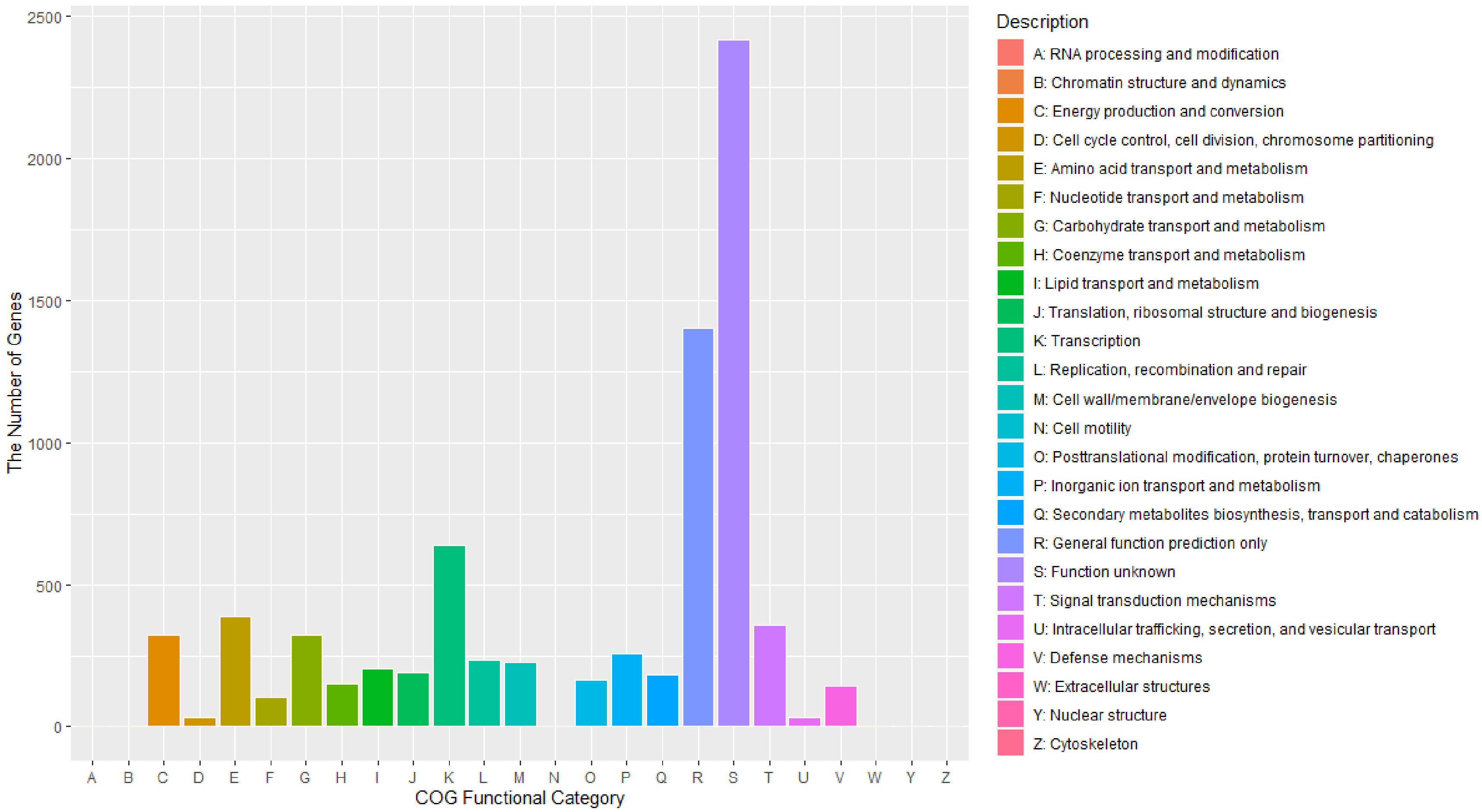

Supplementary Figure S1. COG functional annotation of the predicted proteins in the strain JCM 3382. A total of 7,697 protein-coding genes were annotated and assigned into 23 functional clusters. The x-axis indicates the functional categories shown on the right, and y-axis shows the number of genes.

(A)

#	Match	Entry	Time	Name	ID	ID Name
1	975.099	863	12.51	JBIR-34	0	
2	934.107	278	12.51	TETRANGULOL-8-METHYLET	0	
3	924.532	814	15.39	andrimid	0	andrimid
4	919.536	667	5.77	2-ACETYLAMINOBENZOESAE	0	
5	902.284	815	14.72	moiramide C	0	moiramide C
6	897.926	718	9.66	fumitr C from SIOCNF895	0	fumitr C
7	893.222	74	7.07	TU 4042-3	0	
8	892.976	776	16.55	Nereus3114.457	0	Nereus3114.457
9	892.168	423	12.84	ANTIMYCIN A RT 12.83	0	
10	891.300	424	13.43	ANTIMYCIN A RT 13.43	0	

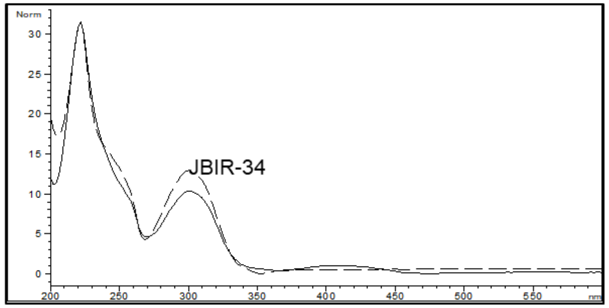

(B)

#	Match	Entry	Time	Name	ID	ID Name
1	982.055	781	16.86	Strevertene A	0	
2	957.028	499	7.33	LAGOSIN	0	
3	945.192	90	10.84	TRIACSIN D (WS 1228 B, TU 6	0	
4	885.470	507	7.97	FILIPIN	0	
5	878.156	549	6.69	NN 21702-Ia	0	
6	869.020	262	9.19	SIMOCYCLINON C4	0	
7	834.739	261	8.37	SIMOCYCLINON C2	0	
8	806.082	509	7.65	PENTAMYCIN	0	
9	761.350	28	2.30	AZOMYCIN (= 2-NITROIMIDAZ	0	
10	740.540	510	10.03	CALBISTRIN A	0	

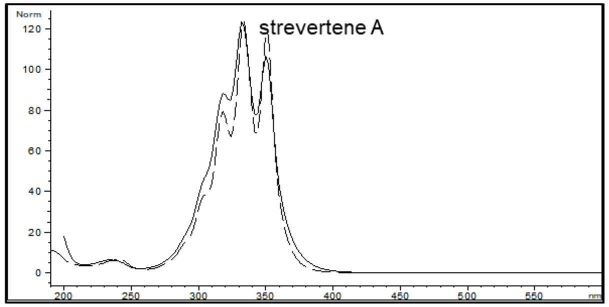

(C)

#	Match	Entry	Time	Name	ID	ID Name
1	955.870	52	11.20	SETOMIMYCIN	0	
2	801.967	212	9.92	XANTHOMEGNIN	0	
3	800.986	834	28.91	6,8-di-O-methylaverufin	0	6,8-di-O-methylaverufin
4	794.614	827	13.97	pannorin	0	pannorin
5	789.585	93	10.65	SPIROFUNGIN (TU 4113-3B)	0	
6	789.063	690	17.22	Antibiotic SS 43405E from 7818	0	Antibiotic SS 43405E
7	781.377	759	12.87	Roridins from 8535.01.BT	0	Roridins
8	779.985	681	12.24	Protorifamycin	0	
9	778.074	225	9.44	FRIDAMYCIN E	0	
10	776.121	835	16.35	4-methoxy-9-methyl-6,7,9,11-tetr	0	4-methoxy-9-methyl-6,7,9,11-tetr

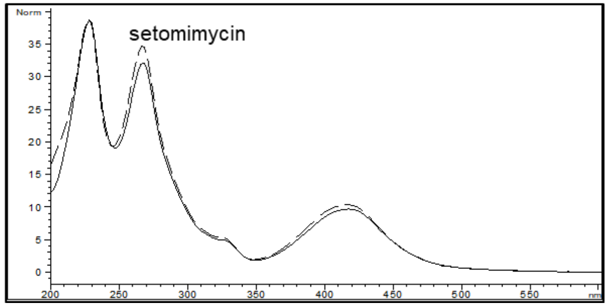

Supplementary Figure S2. In-house UV library screening results for three identified molecules from a culture extract of JCM 3382. A combinatorial analysis of spectral data including UV, LC-HR-MS, and ^{1}H NMR identified each molecule as to tambromycin A (A), linearmycin A (B), and setomimycin (C), respectively.

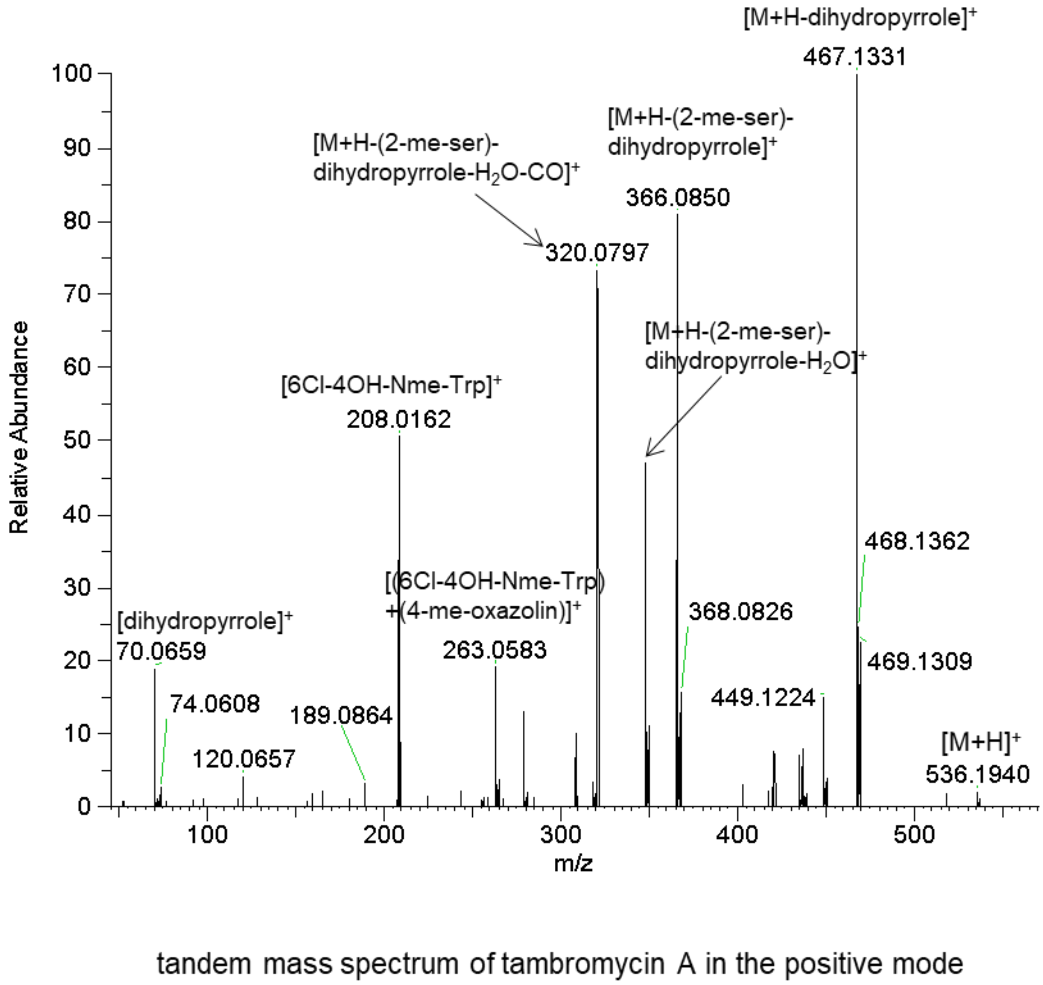

Supplementary Figure S3. Tandem mass spectrum of tambromycin A in the positive mode, and the assignment of fragment ions.

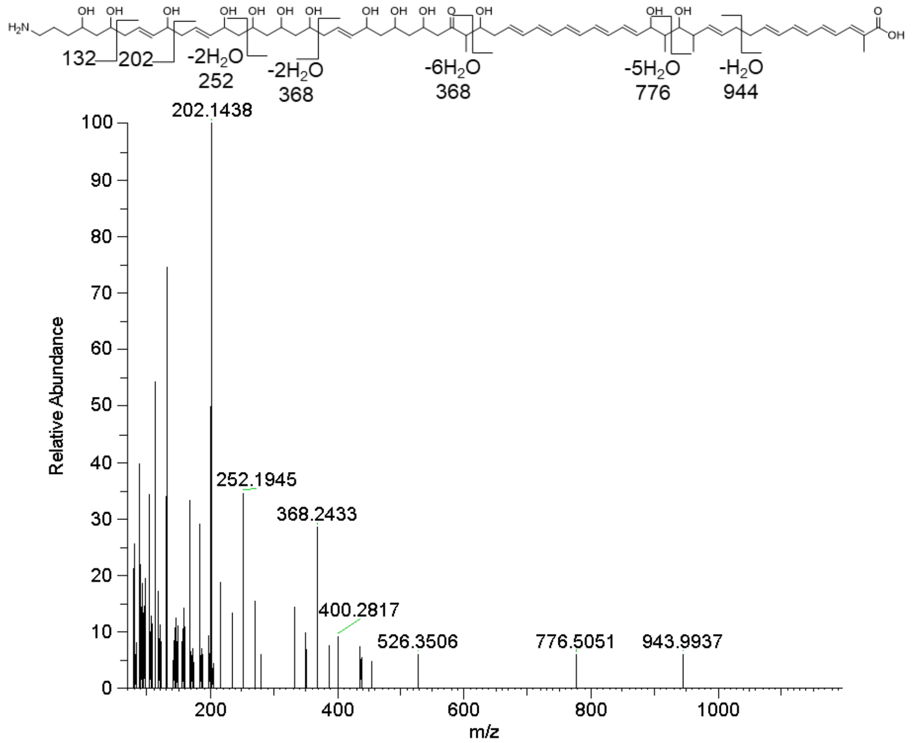

tandem mass spectrum of linearmycin A in the positive mode

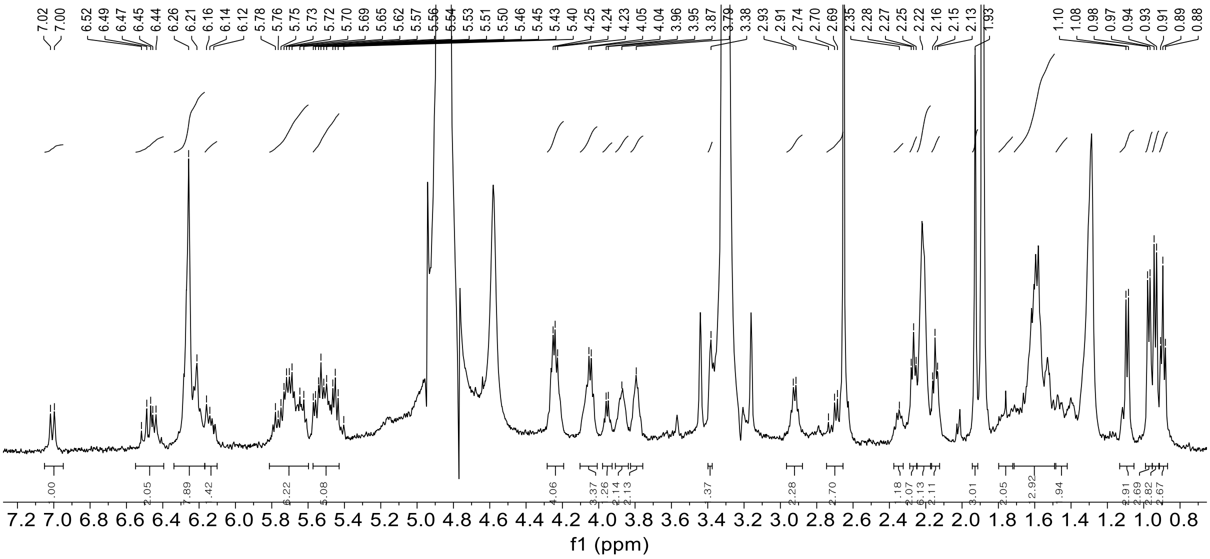

^{1}H NMR (500 MHz, CD_3OD) δ 7.01 (d, *J* = 10.8 Hz, 1H), 6.52 – 6.40 (m, 2H), 6.32-6.18 (m, 10H), 6.15 (dd, *J* = 15.4, 9.1 Hz, 1H), 5.84 – 5.61 (m, 6H), 5.60 – 5.40 (m, 5H), 4.30 – 4.19 (m, 4H), 4.11-4.01 (m, 3H), 3.95 (q, *J* = 5.9 Hz, 1H), 3.87 (m, 2H), 3.79 (m, 2H), 3.37 (m, 1H), 2.92 (d, *J* = 7.4 Hz, 2H), 2.74 – 2.68 (m, 3H), 2.35 (m, 1H), 2.27 (t, *J* = 6.8 Hz, 2H), 2.25-2.18 (m, 8H), 2.15 (t, *J* = 6.9 Hz, 2H), 1.93 (s, 3H), 1.80-1.72 (m, 2H), 1.70-1.50 (m. 13 H), 1.48-1.44 (m. 2H), 1.09 (d, *J* = 6.9 Hz, 3H), 0.97 (d, *J* = 6.7 Hz, 3H), 0.94 (d, *J* = 6.8 Hz, 3H), 0.89 (t, *J* = 6.7 Hz, 2H).

Supplementary Figure S4. MS/MS (upper) and ^{1}H NMR data (bottom) of linearmycin A isolated from JCM 3382.

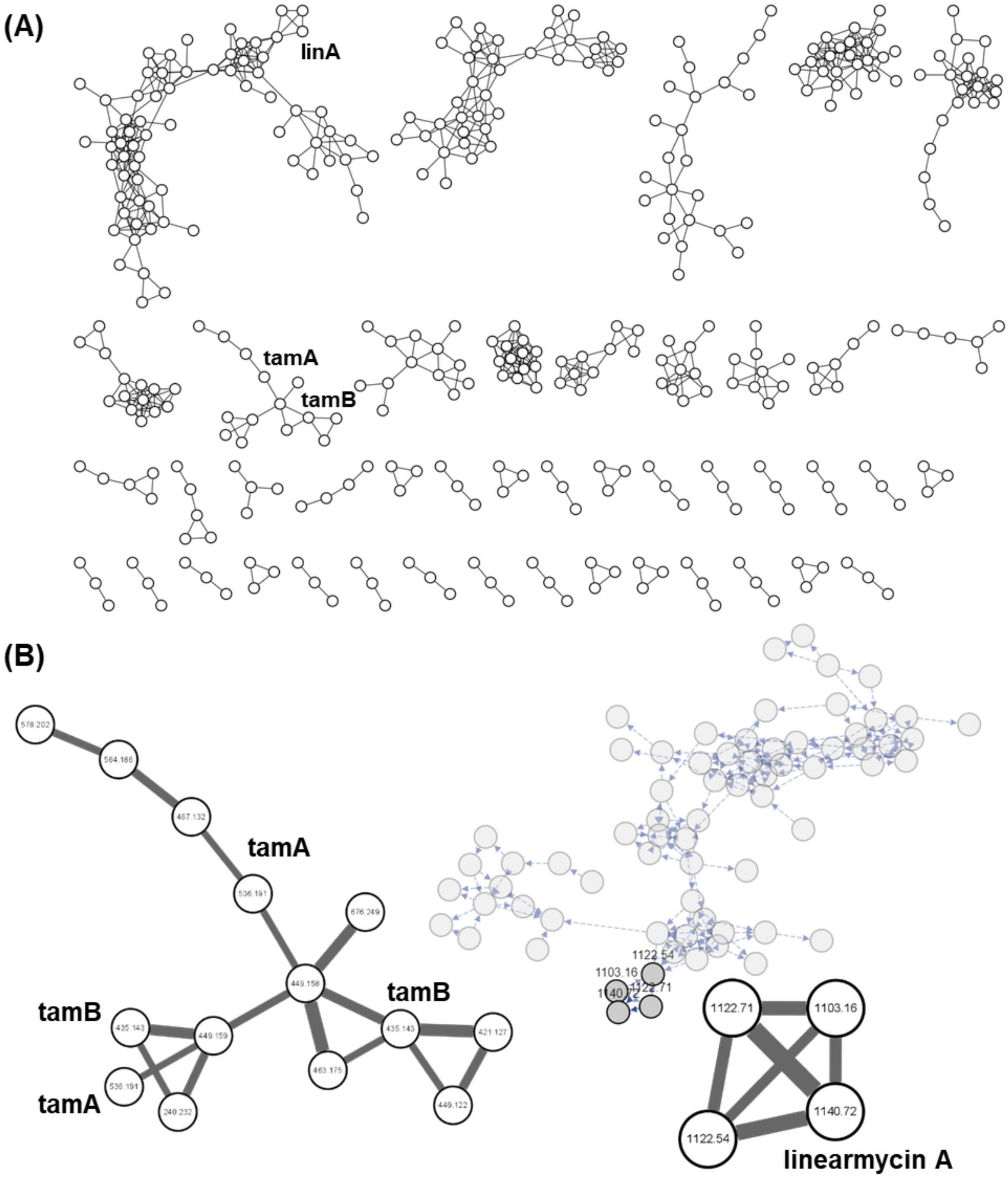

Supplementary Figure S5. Molecular networks constructed with LC-MS/MS data from the culture extract produced by JCM 3382. (A) Partial molecular networks derived from the culture extract of JCM 3382. Self-loops and two-node networks were removed. (B) Clusters from the molecular network of the identified tambromycin (left) and linearmycin (right). Each node represents *m/z* value of the parent ion and edge thickness displays cosine score similarity.

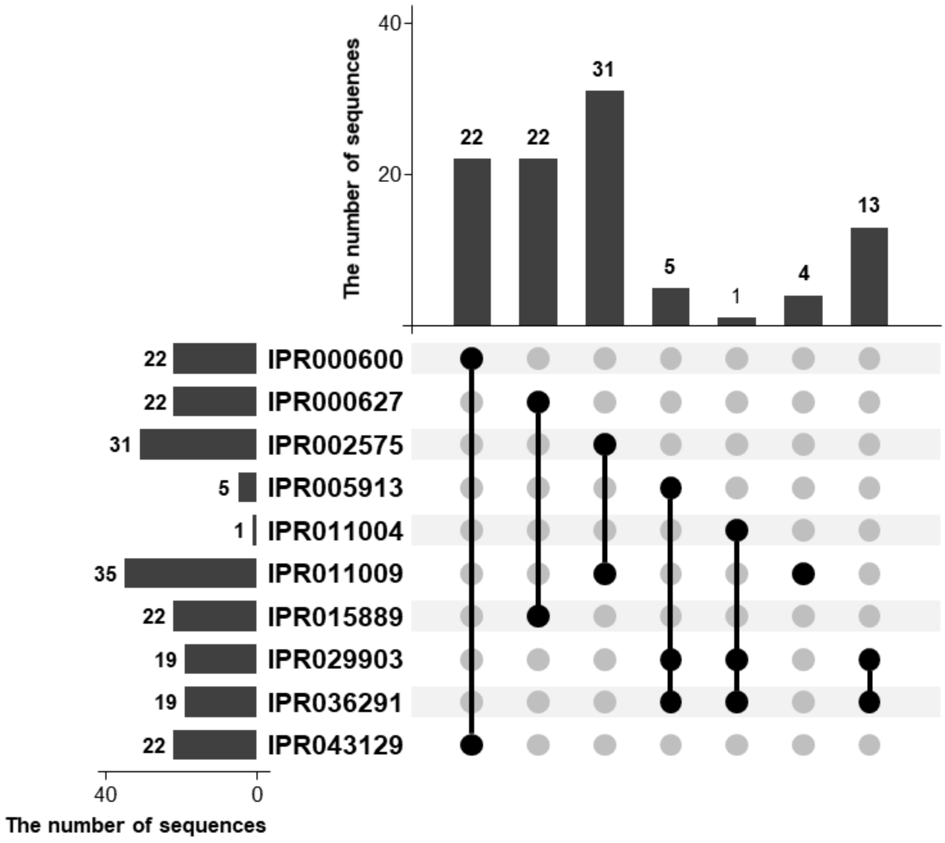

Supplementary Figure S6. Distribution of protein sequences encoded by 98 interleaved genes in disordered clusters by domain profiles. The plot represents intersections between sets within a protein domain matrix and the number of non-zero occurences of those intersections as a bar chart. The set intersection matrix represents presences of protein domains predicted by InterProScan and the number of their occurrences in the 98 proteins are shown as a bar chart. Descriptions for InterPro accessions: ROK (Repressor, ORF, Kinase) family (IPR000600), intradiol ring-cleavage dioxygenase, C-terminal (IPR000627), aminoglycoside phosphotransferase (IPR002575), dTDP-4-dehydrorhamnose reductase family (IPR005913), trimeric LpxA-like superfamily (IPR011004), protein kinase-like domain superfamily (IPR011009), intradiol ring-cleavage dioxygenase, core (IPR015889), RmlD-like substrate binding domain (IPR029903), NAD(P)-binding domain superfamily (IPR036291), and ATPase, nucleotide binding domain (IPR043129).

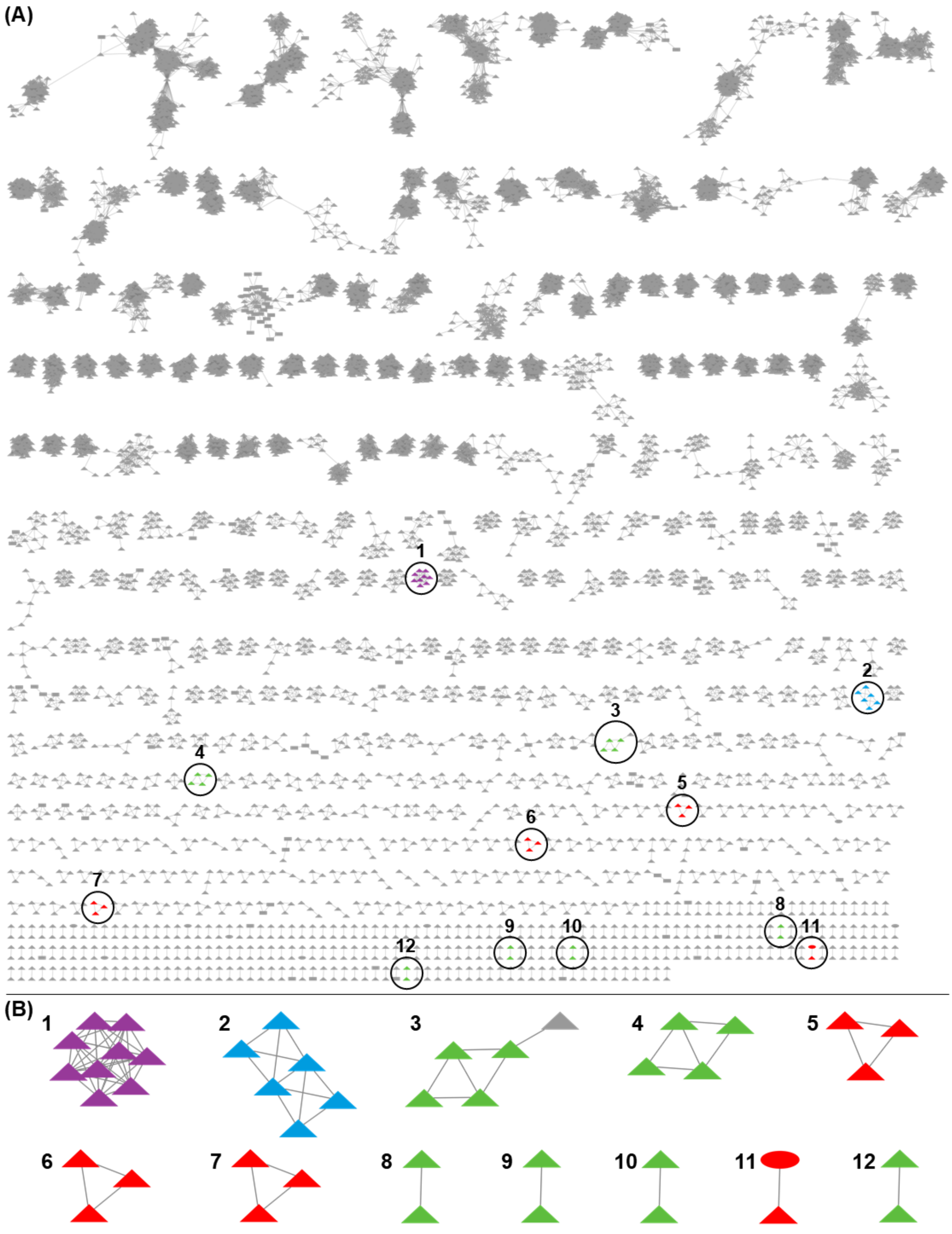

Supplementary Figure S7. Visualization of BGC networks generated by BiG-SCAPE.

(A) Sequence similarity networks of antiSMASH-predicted BGCs containing *gabT1*, *yktC1*, and *gutB1* orthologs in the 110 genomes (Supplementary Table S8) and BGCs in the MIBiG database. Circular, rectangular, and triangular nodes indicate BGCs from JCM 3382, MIBiG,

and the other 109 genomes, respectively. The 42 BGCs (nodes) containing *gabT1*, *yktC1*, and *gutB1* were marked with colors based on gene structures: red for canonical, green for disordered Type 1, purple for disordered Type 2, blue for disordered Type 3, and grey for BGCs not containing orthologs of the three genes. The 12 networks harboring the 42 nodes were marked by circles. (B) A magnified view of the 12 networks harboring the 42 antiSMASH-predicted BGCs containing *gabT1*, *yktC1*, and *gutB1* orthologs.

Supplementary Tables S1, S3-S5 and S7 were attached as a separate file.

Supplementary Table S1. List of 2,061 *Streptomyces* genomes used in the comparative analysis (attached as a separate file).

Supplementary Table S2. Reference gene clusters of tambromycin, setomimycin, and linearmycin.

Gene Cluster*	Identifier*	Position*	Product*
	IF33_RS0114760	141432 - 142709 (-)	cation/H(+) antiporter
	IF33_RS0114765	142696 - 143238 (-)	flavin reductase family protein
	IF33_RS0114770	143401 - 145002 (-)	tryptophan 7-halogenase
	IF33_RS0114775	145178 - 146176 (+)	hypothetical protein
	IF33_RS0114780	146304 - 147815 (+)	aldehyde dehydrogenase family protein
	IF33_RS0114785	147812 - 149644 (+)	non-ribosomal peptide synthetase
	IF33_RS0114790	149648 - 150394 (+)	thioesterase
	IF33_RS0114795	150435 - 150614 (+)	MbtH family NRPS accessory protein
	IF33_RS0114800	150616 - 151830 (+)	cytochrome P450
	IF33_RS0114805	151846 - 155784 (+)	non-ribosomal peptide synthetase
	IF33_RS36805	155784 - 156428 (+)	4'-phosphopantetheinyl transferase superfamily protein
	IF33_RS0114815	156441 - 157472 (+)	winged helix DNA-binding domain-containing protein
Tambromycin	IF33_RS0114820	157592 - 158869 (+)	MFS transporter
(NZ_JOIB01000012.1)	IF33_RS0114825	158897 - 163132 (+)	non-ribosomal peptide synthetase
	IF33_RS0114830	163044 - 166481 (+)	non-ribosomal peptide synthetase
	IF33_RS0114835	166481 - 167887 (+)	hypothetical protein
	IF33_RS0114840	167967 - 169157 (+)	hypothetical protein
	IF33_RS0114845	169154 - 170299 (+)	hypothetical protein
	IF33_RS0114850	170368 - 170949 (-)	GNAT family N-acetyltransferase
	IF33_RS0114855	170976 - 172040 (-)	threonine aldolase
	IF33_RS0114860	172043 - 172765 (-)	DUF2461 domain-containing protein
	IF33_RS0114865	172729 - 174129 (-)	serine hydroxymethyltransferase
	IF33_RS0114870	174184 - 177474 (-)	non-ribosomal peptide synthetase
	IF33_RS0114875	177565 - 179439 (-)	AAA family ATPase
	IF33_RS0114880	179641 - 179847 (+)	hypothetical protein
	IF33_RS0114885	179976 - 183020 (-)	tetratricopeptide repeat protein

	IF33_RS0114890	183161 - 183850 (-)	winged helix-turn-helix transcriptional regulator
Setomimycin (NZ_AOPZ01000074.1)	STRAU_RS10340	2057 - 3571 (+)	DHA2 family efflux MFS transporter permease subunit
	STRAU_RS38955	3603 - 4262 (+)	TcmI family type II polyketide cyclase
	STRAU_RS10350	4754 - 5182 (+)	nuclear transport factor 2 family protein
	STRAU_RS10355	5241 - 6050 (+)	response regulator transcription factor
	STRAU_RS10360	6151 - 7371 (+)	cytochrome P450
	STRAU_RS10365	7383 - 7577 (+)	ferredoxin
	STRAU_RS10370	7645 - 8115 (-)	nuclear transport factor 2 family protein
	STRAU_RS41390	8333 - >8620 (+)	TcmI family type II polyketide cyclase
	STRAU_RS41395	8651 - 8983 (+)	TcmI family type II polyketide cyclase
	STRAU_RS10380	8980 - 10251 (+)	beta-ketoacyl-[acyl-carrier-protein] synthase family protein
	STRAU_RS10385	10248 - 11444 (+)	ketosynthase chain-length factor
	STRAU_RS10390	11507 - 11773 (+)	acyl carrier protein
	STRAU_RS10395	11863 - 12648 (+)	SDR family NAD(P)-dependent oxidoreductase
	STRAU_RS10400	12682 - 13647 (+)	polyketide synthase
	STRAU_RS10405	13644 - 14558 (+)	alpha/beta hydrolase
Linearmycin (CP011664.1)	lnyT	1061319 - 1062308 (-)	agmatinase
	lnyS	1062415 - 1063971 (-)	methylmalonyl-CoA carboxyltransferase
	lnyR	1064457 - 1067375 (+)	transcriptional regulator
	lnyQ	1067356 - 1068252 (+)	metallophosphoesterase
	lnyP	1068249 - 1068992 (+)	4'-phosphopantetheinyl transferase
	lnyO	1069184 - 1070845 (+)	amine oxidase
	lnyN	1070939 - 1072351 (-)	acyl--CoA ligase
	lnyM	1072435 - 1073028 (-)	thioesterase
	lnyL	1073166 - 1073738 (-)	glucose-1-phosphate thymidylyltransferase
	lnyK	1074063 - 1075127 (+)	daunorubicin ABC transporter ATPase
	lnyJ	1075226 - 1075996 (+)	ABC transporter
	lnyI	1076160 - 1077197 (+)	ACP S-malonyltransferase
	lnyHI	1077302 - 1094107 (-)	polyketide synthase
	lnyHH	1094175 - 1116644 (-)	polyketide synthase
	lnyHG	1116707 - 1126318 (-)	polyketide synthase
	lnyHF	1126413 - 1142603 (-)	polyketide synthase
	lnyHE	1142689 - 1158345 (-)	polyketide synthase
	lnyHD	1158599 - 1163497 (-)	polyketide synthase
	lnyHC	1163620 - 1183653 (-)	polyketide synthase

lnyHB	1183678 - 1193985 (-)	polyketide synthase
lnyHA	1194150 - 1228586 (-)	polyketide synthase
lnyG	1228902 - 1230452 (-)	membrane protein
lnyF	1230449 - 1230949 (-)	membrane protein
lnyE	1231012 - 1231545 (-)	LnyE
lnyD	1231711 - 1233006 (+)	histidine kinase
lnyC	1233071 - 1233631 (+)	LuxR family transcriptional regulator
lnyB	1233701 - 1236565 (-)	transcriptional regulator

[*] Gene cluster information including identifier, position, and product was obtained from the annotation of each genome.

Supplementary Table S3. TBLASTN and BLASTN results of the 27 proteins and the whole nucleotide sequences of the tambromycin gene cluster in the 2,062 *Streptomyces* genomes (attached as a separate file). Cells were filled with -1 if there was no hits (E-value < $1e^{-5}$). BLASTN column shows sum of significant hits (E-value < $1e^{-20}$).

Supplementary Table S4. TBLASTN and BLASTN results of the 15 proteins and the whole nucleotide sequences of the setomimycin gene cluster in the 2,062 *Streptomyces* genomes (attached as a separate file). Cells were filled with -1 if there was no hits (E-value < $1e^{-5}$). BLASTN column shows sum of significant hits (E-value < $1e^{-20}$).

Supplementary Table S5. TBLASTN and BLASTN results of the 27 proteins and the whole nucleotide sequences of the linearmycin gene cluster in the 2,062 *Streptomyces* genomes (attached as a separate file). Cells were filled with -1 if there was no hits (E-value < $1e^{-5}$). BLASTN column shows sum of significant hits (E-value < $1e^{-20}$).

Supplementary Table S6. List of putative antibiotics gene clusters found in 2,062 *Streptomyces* genomes.

Species	Genome Accession	Location	Length (bp)
Tambromycin			
Streptomyces sp. PCS3-D2	GCF_000612545.1	NZ_JDUZ01000004.1:52213-94727	42,515
Streptomyces sp. NRRL S-118	GCF_000716335.1	NZ_KL591003.1:203159-246093	42,935
Streptomyces virginiae NRRL B-8091	GCF_000716685.1	NZ_JNYC01000001.1:339327-383531	44,205
Streptomyces sp. NRRL S-241	GCF_000717925.1	NZ_JOCY01000002.1:220277-262725	42,449
Streptomyces lavendulae subsp. *lavendulae* NRRL WC-3542	GCF_000718705.1	NZ_JOBU01000006.1:313161-355581	42,421
Streptomyces sp. NRRL S-237	GCF_000718935.1	NZ_JODA01000002.1:213507-255337	41,831
Streptomyces sp. NRRL S-575	GCF_000718955.1	NZ_JODJ01000013.1:66572-108248	41,677
Streptomyces sp. NRRL F-2664	GCF_000719345.1	NZ_JOFX01000001.1:38864-81127	42,264
Streptomyces sp. NRRL B-1322	GCF_000719625.1	NZ_JOHF01000024.1:37672-80092	42,421
Streptomyces sp. NRRL F-4474	GCF_000719855.1	NZ_JOIB01000012.1:141435-183850	42,416
Streptomyces sp. NRRL S-98	GCF_000720235.1	NZ_JOJD01000002.1:535243-577766	42,524
Streptomyces virginiae NRRL ISP-5094	GCF_000720455.1	NZ_JOAK01000003.1:37572-80092	42,521
Streptomyces sp. NRRL S-515	GCF_000720795.1	NZ_JODE01000010.1:88791-130467	41,677
Streptomyces erythrochromogenes NRRL B-2112	GCF_000725555.1	NZ_KL647064.1:185826-228264	42,439
Streptomyces yangpuensis fd2-tb	GCF_001005295.1	NZ_LBMK01000003.1:806704-849341	42,638
Streptomyces sp. WM6349	GCF_001279445.1	NZ_LGCZ01000185.1:37647-80067	42,421
Streptomyces sp. XY511	GCF_001279485.1	NZ_LGDM01000305.1:43323-85767	42,445
Streptomyces sp. WM6368	GCF_001279625.1	NZ_LGDA01000013.1:60923-103569	42,647
Streptomyces sp. WM6373	GCF_001279685.1	NZ_LGDC01000072.1:38035-80570	42,536
Streptomyces sp. XY413	GCF_001279765.1	NZ_LGDP01000222.1:94763-137283	42,521
Streptomyces sp. XY37	GCF_001279815.1	NZ_LGDN01000168.1:103688-146224	42,537
Streptomyces sp. XY58	GCF_001279855.1	NZ_LGDK01000220.1:37797-80333	42,537
Streptomyces sp. XY593	GCF_001279885.1	NZ_LGDJ01000013.1:37708-80167	42,460
Streptomyces sp. XY66	GCF_001279905.1	NZ_LGDI01000013.1:37766-80287	42,522
Streptomyces sp. H036	GCF_001279925.1	NZ_LGDS01000024.1:109059-151479	42,421
Streptomyces sp. MMG1064	GCF_001279965.1	NZ_LGDT01000206.1:103798-146334	42,537
Streptomyces sp. IMTB 1903	GCF_001531145.1	NZ_LQYB01000013.1:35453-77902	42,450
Streptomyces sp. TN58	GCF_001941845.1	NZ_CP018870.1:857610-900070	42,461
Streptomyces sp. fd1-xmd	GCF_002007685.1	NZ_CP019798.1:1158549-1200947	42,399
Streptomyces sp. 3211	GCF_002028385.1	NZ_CP020039.1:1231297-1273850	42,554
Streptomyces sp. Sge12	GCF_002080455.1	NZ_CP020555.1:6970858-7013455	42,598

Streptomyces sp. CG 926	GCF_003148825.1	NZ_QGGZ01000006.1:107394-149293	41,900
Streptomyces sp. A0592	GCF_004795115.1	NZ_SSBM01000012.1:100800-143232	42,433
Streptomyces sp. ICN19	GCF_005795905.1	NZ_VAWE01000001.1:7591980-7634063	42,084
Streptomyces sp. TRM49041	GCF_008271805.1	NZ_VSRY01000158.1:64625-106639	42,015
Streptomyces venezuelae ATCC 21018	GCF_008642275.1	NZ_CP029189.1:673646-715867	42,222
Streptomyces subrutilus ATCC 27467	GCF_008704535.1	NZ_CP023701.1:7179706-7222364	42,659
Streptomyces sp. gCLA4	GCF_014170455.1	NZ_MBDH01000012.1:43035-85553	42,519
Streptomyces sp. 3211.5	GCF_014203415.1	NZ_JACHIX010000005.1:336961-379515	42,555
Streptomyces sp. 3211.3	GCF_014203435.1	NZ_JACHIY010000003.1:658702-701256	42,555
Streptomyces sp. INR7	GCF_014217585.1	NZ_CP043638.1:6933598-6976033	42,436
Streptomyces nojiriensis JCM 3382 (draft)	GCF_014648615.1	NZ_BMRL01000002.1:242639-284365	41,727
Streptomyces avidinii JCM 4726	GCF_014650695.1	NZ_BMVL01000003.1:158369-200781	42,413
Streptomyces subrutilus JCM 4834	GCF_014650935.1	NZ_BMVX01000011.1:16195-58853	42,659
Streptomyces wuyuanensis CGMCC 4.7042	GCF_900103455.1	NZ_FNHI01000008.1:233417-275569	42,153
Streptomyces nojiriensis JCM 3382 (complete; this study)	GCF_017639205.1	CP071139.1:1575763-1617489	41,727
<u>Setomimycin</u>			
Streptomyces aurantiacus JA 4570	GCF_000414115.1	NZ_AOPZ01000074.1:2057-14555	12,499
Streptomyces sp. NRRL F-2580	GCF_000720115.1	NZ_JOIR01000032.1:36176-48571	12,396
Streptomyces sp. NRRL F-5755	GCF_001279365.1	NZ_LGCW01000311.1:22254-35024	12,771
Streptomyces sp. GKU 895	GCF_002027195.1	NZ_MWJO01000012.1:73715-86348	12,634
Streptomyces sp. CG 926	GCF_003148825.1	NZ_QGGZ01000005.1:123177-135652	12,476
Streptomyces aquilus GGCR-6	GCF_003955715.1	NZ_CP034463.1:6759729-6772307	12,579
Streptomyces sp. BK042	GCF_004343295.1	NZ_SLZH01000003.1:409440-422019	12,580
Streptomyces nojiriensis JCM 3382 (draft)	GCF_014648615.1	NZ_BMRL01000003.1:130644-143093	12,450
Streptomyces sp. 3R004	GCF_015163075.1	NZ_JADDXU010000005.1:31138-43800	12,663
Streptomyces sp. TLI_55	GCF_900206255.1	NZ_OAOJ01000001.1:6022130-6034735	12,606
Streptomyces nojiriensis JCM 3382 (complete; this study)	GCF_017639205.1	CP071139.1:4304900-4317349	12,450
<u>Linearmycin</u>			
Streptomyces sp. Mg1	GCF_000412265.2	NZ_CP011664.1:1061322-1236565	175,244
Streptomyces sp. CB03578	GCF_001905025.1	NZ_LWLD01000001.1:228979-404181	175,203
Streptomyces lavendulae subsp. *lavendulae* CCM 3239	GCF_002803845.1	NZ_CP024985.1:1804334-1980268	175,935
Streptomyces sp. ADI95-16	GCF_003721495.1	NZ_CP033581.1:902068-1077345	175,278
Streptomyces venezuelae ATCC 21018	GCF_008642275.1	NZ_CP029189.1:1023581-1198949	175,369
Streptomyces nojiriensis JCM 3382 (complete; this study)	GCF_017639205.1	CP071139.1:7844799-8020177	175,379

Supplementary Table S7. TBLASTN and BLASTN results of the three proteins and the whole nucleotide sequences of the putative *gabT1-yktC1-gutB1* gene cluster in the 2,062 *Streptomyces* genomes (attached as a separate file).

Supplementary Table S8. List of 114 putative *gabT1-yktC1-gutB1* gene clusters found in 2,062 *Streptomyces* genomes.

Species	Genome Accession	Category	Type	Location	Length (bp)
Streptomyces albofaciens JCM 4342	GCF_008634025.1	Canonical		NZ_PDCM01000002.1:2066071-2069329	3,259
Streptomyces albus subsp. *albus* NRRL F-4371	GCF_001541145.1	Canonical		NZ_LMZE01000193.1:8884-12127	3,244
Streptomyces capuensis NRRL B-12337	GCF_000716845.1	Canonical		NZ_JNWP01000026.1:34181-37430	3,250
Streptomyces capuensis NRRL B-3501	GCF_000718295.1	Canonical		NZ_JOFM01000025.1:70246-73495	3,250
Streptomyces chrestomyceticus JCM 4735	GCF_003865135.1	Canonical		NZ_BHZC01000001.1:2220294-2223525	3,232
Streptomyces ficellus NRRL 8067	GCF_009739905.1	Canonical		NZ_CP034279.1:1538297-1541528	3,232
Streptomyces griseoflavus NRRL B-1830	GCF_001270585.1	Canonical		NZ_LGUW01000363.1:34182-37425	3,244
Streptomyces lavendulae subsp. *lavendulae* NRRL B-2775	GCF_000721155.1	Canonical		NZ_JOEX01000031.1:34181-37430	3,250
Streptomyces lavendulae subsp. *lavendulae* NRRL WC-3532	GCF_000718855.1	Canonical		NZ_JOCN01000028.1:34288-37537	3,250
Streptomyces malaysiense MUSC 136	GCF_000980885.2	Canonical		NZ_LBDA02000051.1:34379-37625	3,247
Streptomyces monomycini NRRL B-24309	GCF_000715845.1	Canonical		NZ_KL571077.1:142744-145928	3,185
Streptomyces nojiriensis JCM 3382 (complete; this study)	GCF_017639205.1	Canonical		CP071139.1:7657371-7660614	3,244
Streptomyces nojiriensis JCM 3382 (draft)	GCF_014648615.1	Canonical		NZ_BMRL01000010.1:150415-153658	3,244
Streptomyces orinoci NRRL B-3379	GCF_003121295.1	Canonical		NZ_PHNC01000010.1:1957-5188	3,232
Streptomyces peucetius NRRL WC-3868	GCF_000725565.1	Canonical		NZ_JOCK01000025.1:69051-72303	3,253
Streptomyces piniterrae jys28	GCF_005048155.1	Canonical		NZ_SUMB01000002.1:870644-873890	3,247
Streptomyces rimosus ATCC 10970	GCF_008704655.1	Canonical		NZ_CP023688.1:2142089-2145338	3,250
Streptomyces rimosus M527	GCF_004196335.1	Canonical		NZ_SADA01000175.1:435076-438325	3,250
Streptomyces rimosus R6-500	GCF_000707925.2	Canonical		NZ_CP045803.1:4573515-4576764	3,250
Streptomyces rimosus R6-500MV9	GCF_000707945.1	Canonical		NZ_JMGX01000025.1:52216-55465	3,250
Streptomyces rimosus R6-500MV9-R8	GCF_000707965.1	Canonical		NZ_JMGY01000014.1:138336-141585	3,250
Streptomyces rimosus subsp. *paromomycinus* NBRC 15454	GCF_003865155.1	Canonical		NZ_BHZD01000001.1:2363543-2366777	3,235
Streptomyces rimosus subsp. *pseudoverticillatus* NRRL WC-3896	GCF_001279345.1	Canonical		NZ_LGCV01000061.1:41930-45182	3,253
Streptomyces rimosus subsp. *rimosus* ATCC 10970	GCF_000331185.1	Canonical		NZ_ANSJ01000025.1:34250-37499	3,250
Streptomyces rimosus subsp. *rimosus* NRRL B-16073	GCF_000716515.1	Canonical		NZ_JNWX01000044.1:9359-12611	3,253
Streptomyces rimosus subsp. *rimosus* NRRL B-2626	GCF_000721045.1	Canonical		NZ_JOEO01000026.1:34145-37394	3,250
Streptomyces rimosus subsp. *rimosus* NRRL B-2660	GCF_000719185.1	Canonical		NZ_JOES01000079.1:34182-37425	3,244

Streptomyces rimosus subsp. *rimosus* NRRL B-8076	GCF_000716745.1	Canonical	NZ_JNYK01000024.1:52104-55353	3,250
Streptomyces rimosus subsp. *rimosus* NRRL ISP-5260	GCF_000717285.1	Canonical	NZ_JNYR01000013.1:138361-141610	3,250
Streptomyces rimosus subsp. *rimosus* NRRL WC-3558	GCF_000720605.1	Canonical	NZ_JOBS01000026.1:34235-37484	3,250
Streptomyces rimosus subsp. *rimosus* NRRL WC-3560	GCF_000718755.1	Canonical	NZ_JOBV01000068.1:34182-37425	3,244
Streptomyces rimosus subsp. *rimosus* NRRL WC-3869	GCF_001279065.1	Canonical	NZ_LGCQ01000136.1:62341-65593	3,253
Streptomyces rimosus subsp. *rimosus* NRRL WC-3873	GCF_001279105.1	Canonical	NZ_LGCS01000283.1:66630-69879	3,250
Streptomyces rimosus subsp. *rimosus* NRRL WC-3874	GCF_000718715.1	Canonical	NZ_JOBT01000052.1:125569-128818	3,250
Streptomyces rimosus subsp. *rimosus* NRRL WC-3875	GCF_001279075.1	Canonical	NZ_LGCR01000082.1:34156-37405	3,250
Streptomyces rimosus subsp. *rimosus* NRRL WC-3876	GCF_000718835.1	Canonical	NZ_JOCM01000010.1:138358-141607	3,250
Streptomyces rimosus subsp. *rimosus* NRRL WC-3877	GCF_000720685.1	Canonical	NZ_JOCL01000028.1:34156-37405	3,250
Streptomyces rimosus subsp. *rimosus* NRRL WC-3880	GCF_000718895.1	Canonical	NZ_JOCP01000010.1:138358-141607	3,250
Streptomyces rimosus subsp. *rimosus* NRRL WC-3882	GCF_000718865.1	Canonical	NZ_JOCO01000025.1:34156-37405	3,250
Streptomyces rimosus subsp. *rimosus* NRRL WC-3897	GCF_001507395.1	Canonical	NZ_LMWF01000140.1:34156-37405	3,250
Streptomyces rimosus subsp. *rimosus* NRRL WC-3898	GCF_001279375.1	Canonical	NZ_LGCU01000091.1:34156-37405	3,250
Streptomyces rimosus subsp. *rimosus* NRRL WC-3899	GCF_001279095.1	Canonical	NZ_LGCT01000117.1:34156-37405	3,250
Streptomyces rimosus subsp. *rimosus* NRRL WC-3900	GCF_000720715.1	Canonical	NZ_JOCR01000022.1:34156-37405	3,250
Streptomyces rimosus subsp. *rimosus* NRRL WC-3904	GCF_000720725.1	Canonical	NZ_JOCQ01000037.1:39889-43168	3,280
Streptomyces rimosus subsp. *rimosus* NRRL WC-3909	GCF_001279015.1	Canonical	NZ_LGCO01000093.1:34156-37405	3,250
Streptomyces rimosus subsp. *rimosus* NRRL WC-3924	GCF_000717815.1	Canonical	NZ_JOBW01000056.1:9642-12894	3,253
Streptomyces rimosus subsp. *rimosus* NRRL WC-3925	GCF_000720565.1	Canonical	NZ_JOBN01000025.1:34156-37405	3,250
Streptomyces rimosus subsp. *rimosus* NRRL WC-3927	GCF_000720595.1	Canonical	NZ_JOBO01000036.1:59392-62644	3,253
Streptomyces rimosus subsp. *rimosus* NRRL WC-3929	GCF_000721665.1	Canonical	NZ_JOJJ01000025.1:52281-55533	3,253
Streptomyces rimosus subsp. *rimosus* NRRL WC-3930	GCF_000718675.1	Canonical	NZ_JOBP01000021.1:66268-69520	3,253
Streptomyces rimosus WT5260	GCF_006229535.1	Canonical	NZ_CP025551.1:2140350-2143599	3,250
Streptomyces sp. CB00455	GCF_001905445.1	Canonical	NZ_LIVQ01000007.1:93500-96749	3,250
Streptomyces sp. ICC1	GCF_003287935.1	Canonical	NZ_CP030287.1:6397128-6400410	3,283
Streptomyces sp. ICC4	GCF_003287915.1	Canonical	NZ_CP030286.1:6325253-6328535	3,283
Streptomyces sp. IMTB 2501	GCF_001953885.1	Canonical	NZ_MQUS01000018.1:146248-149491	3,244
Streptomyces sp. NRRL B-11253	GCF_000716455.1	Canonical	NZ_JNWN01000024.1:68966-72218	3,253
Streptomyces sp. NRRL F-5755	GCF_001279365.1	Canonical	NZ_LGCW01000103.1:66428-69668	3,241
Streptomyces sp. NRRL S-237	GCF_000718935.1	Canonical	NZ_JODA01000006.1:146330-149585	3,256
Streptomyces sp. NRRL WC-3701	GCF_001279025.1	Canonical	NZ_LGCP01000128.1:34156-37405	3,250
Streptomyces sp. NRRL WC-3702	GCF_000717805.1	Canonical	NZ_JOCC01000028.1:34156-37405	3,250
Streptomyces sp. NRRL WC-3703	GCF_000718815.1	Canonical	NZ_JOCH01000023.1:34156-37405	3,250
Streptomyces sp. PCS3-D2	GCF_000612545.1	Canonical	NZ_JDUZ01000001.1:878954-882221	3,268
Streptomyces sp. SID1046	GCF_009865565.1	Canonical	NZ_WWKI01000474.1:1647-4935	3,289
Streptomyces sp. SID5471	GCF_009862935.1	Canonical	NZ_WWIL01000267.1:34250-37499	3,250

Streptomyces sp. WAC 06725	GCF_003947585.1	Canonical		NZ_QHJO01000069.1:27357-30606	3,250
Streptomyces sp. WAC 06783	GCF_003947575.1	Canonical		NZ_QHJP01000017.1:30071-33323	3,253
Streptomyces subrutilus ATCC 27467	GCF_008704535.1	Canonical		NZ_CP023701.1:1387089-1390398	3,310
Streptomyces subrutilus JCM 4834	GCF_014650935.1	Canonical		NZ_BMVX01000007.1:221413-224722	3,310
Streptomyces virginiae NRRL B-8091	GCF_000716685.1	Canonical		NZ_JNYC01000003.1:179084-182354	3,271
Streptomyces viridochromogenes NRRL 3413	GCF_001270495.1	Canonical		NZ_LGUR01000240.1:76576-79825	3,250
Streptomyces viridochromogenes NRRL 3414	GCF_001047325.1	Canonical		NZ_LFNT01000035.1:14402-17651	3,250
Streptomyces viridochromogenes NRRL 3416	GCF_001270485.1	Canonical		NZ_LGUQ01000269.1:76575-79824	3,250
Streptomyces zaomyceticus JCM 4864	GCF_014656215.1	Canonical		NZ_BNBZ01000038.1:65874-69054	3,181
Streptomyces goshikiensis JCM 4640	GCF_014650555.1	Disordered	Type1	NZ_BMVE01000015.1:16118-22325	6,208
Streptomyces lavendulae subsp. *lavendulae* CCM 3239	GCF_002803845.1	Disordered	Type1	NZ_CP024985.1:7615317-7621533	6,217
Streptomyces lavendulae subsp. *lavendulae* NRRL B-2774	GCF_000718155.1	Disordered	Type1	NZ_JOEW01000029.1:63911-70127	6,217
Streptomyces niveus NCIMB 11891	GCF_000497425.1	Disordered	Type1	NZ_CM002280.1:4132714-4138636	5,923
Streptomyces niveus NRRL 2449	GCF_001723075.1	Disordered	Type1	NZ_MDCS01000135.1:7094-13016	5,923
Streptomyces niveus NRRL 2466	GCF_001723055.1	Disordered	Type1	NZ_MDCR01000161.1:9497-15400	5,904
Streptomyces nojiriensis JCM 3382 (complete; this study)	GCF_017639205.1	Disordered	Type1	CP071139.1:8060004-8066245	6,242
Streptomyces nojiriensis JCM 3382 (draft)	GCF_014648615.1	Disordered	Type1	NZ_BMRL01000028.1:41356-47597	6,242
Streptomyces sp. 3211.1	GCF_003550235.1	Disordered	Type1	NZ_QXDI01000001.1:8021652-8027898	6,247
Streptomyces sp. 4R-3d	GCF_004563805.1	Disordered	Type1	NZ_SPNN01000014.1:94934-100836	5,903
Streptomyces sp. ADI95-17	GCF_003846175.1	Disordered	Type1	NZ_ML123051.1:126449-132745	6,297
Streptomyces sp. CB02120-2	GCF_002803195.1	Disordered	Type1	NZ_NNBM01000004.1:247996-254194	6,199
Streptomyces sp. CB03578	GCF_001905025.1	Disordered	Type1	NZ_LWLD01000005.1:259729-265948	6,220
Streptomyces sp. LamerLS-316	GCF_002711365.1	Disordered	Type1	NZ_AQRG01000001.1:117820-124017	6,198
Streptomyces sp. MJM1172	GCF_001905425.1	Disordered	Type1	NZ_LIVP01000021.1:61422-67629	6,208
Streptomyces sp. NRRL F-2747	GCF_000720145.1	Disordered	Type1	NZ_JOIS01000028.1:15565-21818	6,254
Streptomyces sp. SID4921	GCF_009863285.1	Disordered	Type1	NZ_WWJL01000017.1:117820-124017	6,198
Streptomyces sp. TSRI0281	GCF_001905125.1	Disordered	Type1	NZ_LWLE01000012.1:669677-675825	6,149
Streptomyces sp. WAC01280	GCF_003949845.1	Disordered	Type1	NZ_RPSA01000001.1:290711-296848	6,138
Streptomyces sp. WM6372	GCF_001279655.1	Disordered	Type1	NZ_LGDB01000222.1:28342-34600	6,259
Streptomyces subrutilus ATCC 27467	GCF_008704535.1	Disordered	Type1	NZ_CP023701.1:7031983-7038227	6,245
Streptomyces subrutilus JCM 4834	GCF_014650935.1	Disordered	Type1	NZ_BMVX01000032.1:25851-32095	6,245
Streptomyces autolyticus CGMCC0516	GCF_001983975.1	Disordered	Type2	NZ_CP019458.1:7026420-7032082	5,663
Streptomyces malaysiensis DSM 14702	GCF_011800555.1	Disordered	Type2	NZ_JAALLH010000001.1:7208646-7214278	5,633
Streptomyces malaysiensis DSM 4137	GCF_002591335.1	Disordered	Type2	NZ_CP023992.1:6209859-6215455	5,597
Streptomyces malaysiensis DSM 4137	GCF_006974005.1	Disordered	Type2	NZ_CP029823.1:4479036-4484632	5,597
Streptomyces malaysiensis F913	GCF_002891865.1	Disordered	Type2	NZ_LJIW01000002.1:4511691-4517320	5,630
Streptomyces samsunensis SA31	GCF_013345665.1	Disordered	Type2	NZ_JABUXT010000037.1:17184-22936	5,753

Streptomyces sp. GP55	GCF_002846355.1	Disordered	Type2	NZ_PJMT01000001.1:7900321-7905660	3,259
Streptomyces sp. M56	GCF_002812405.1	Disordered	Type2	NZ_CP025018.1:4544053-4549573	3,244
Streptomyces sp. NRRL F-6131	GCF_000719695.1	Disordered	Type2	NZ_JOHN01000011.1:61886-67275	3,250
Streptomyces sp. SID8382	GCF_009864805.1	Disordered	Type2	NZ_WWFZ01000394.1:13400-18920	3,250
Streptomyces sp. SPMA113	GCF_001748165.1	Disordered	Type2	NZ_BDFA01000014.1:158733-164311	3,232
Streptomyces sulfonofaciens JCM 5069	GCF_014656295.1	Disordered	Type2	NZ_BNCD01000028.1:45030-50500	3,232
Streptomyces violaceorubidus NRRL B-16381	GCF_000717995.1	Disordered	Type2	NZ_JODM01000039.1:21697-27156	3,244
Streptomyces auratus AGR0001	GCF_000280865.1	Disordered	Type3	NZ_JH725389.1:260139-265512	3,250
Streptomyces sioyaensis DSM 40032	GCF_004122735.1	Disordered	Type3	NZ_SDIF01000006.1:36638-42010	3,250
Streptomyces sp. 2112.2	GCF_900104865.1	Disordered	Type3	NZ_FNSG01000001.1:1199290-1204663	3,247
Streptomyces sp. 2314.4	GCF_900105525.1	Disordered	Type3	NZ_FNSJ01000001.1:1204572-1209945	3,185
Streptomyces sp. 2333.5	GCF_002797835.1	Disordered	Type3	NZ_PGEO01000001.1:1197569-1202942	3,244
Streptomyces sp. TM32	GCF_004122755.1	Disordered	Type3	NZ_SDIG01000002.1:79187-84558	3,244
